# Simulating thoughts to measure and study internal attention in mental health

**DOI:** 10.1038/s41598-021-81756-w

**Published:** 2021-01-26

**Authors:** Iftach Amir, Liad Ruimi, Amit Bernstein

**Affiliations:** Observing Minds Lab, School of Psychological Sciences, University of Haifa, Mount Carmel, 31905 Haifa, Israel

**Keywords:** Attention, Cognitive control, Anxiety, Depression, Emotion

## Abstract

Our mind’s eye and the role of internal attention in mental life and suffering has intrigued scholars for centuries. Yet, experimental study of internal attention has been elusive due to our limited capacity to control the timing and content of internal stimuli. We thus developed the Simulated Thoughts Paradigm (STP) to experimentally deliver own-voice thought stimuli that simulate the content and experience of thinking and thereby experimental study of internal attentional processes. In independent experiments (N = 122) integrating STP into established cognitive-experimental tasks, we found and replicated evidence that emotional reactivity to negative thoughts predicts difficulty disengaging internal attention from, as well as biased selective internal attention of, those thoughts; these internal attention processes predict cognitive vulnerability (e.g., negative repetitive thinking) which thereby predict anxiety and depression. Proposed methods and findings may have implications for the study of information processing and attention in mental health broadly and models of internal attentional (dys)control in cognitive vulnerability and mental health more specifically.

## Introduction

To adapt, survive and thrive, our minds are continuously challenged to process and respond to events in our external (i.e., sensory) and internal (i.e., mental) environments^1–3^. The brain’s solution to this ever-present challenge is attention—the selection and modulation of sources and objects of external and internal information^4,5^. Accordingly, dysregulation of attentional processes has long been implicated in information processing theories of prevalent mental disorders such as mood and anxiety disorders^6–8^. Yet research to-date has focused almost exclusively on dysregulation of *external* attention^7,9^. Much less research has focused on dysregulation of *internal* attention broadly or in mental health specifically^1,3^. We argue that (dys)regulation of *internal* attention as well as the dynamic interplay between *internal* and *external* attention, may be fundamentally important to (mal)adaption and mental health. Furthermore, we argue that efforts to more directly study *internal* attention may prove critical to long-sought basic and clinical science of information processing in mental health.

### External attention (dys)regulation

*External attention* is the processing of perceptual-sensory information incoming from a source external to the mind/brain such as the peripheral nervous system, originating from outside and/or within the body (e.g., visual information incoming via the eyes, proprioceptive sensations originating from the muscles)^4,10,11^. Theory and data suggest that external attentional processes are governed by two interacting yet distinct attentional systems—the stimulus-driven (bottom-up) and goal-directed (top-down) systems^4,12^. Dysregulation in the goal-directed system is theorized to drive (maladaptive) strategic avoidance of- or difficulties in disengaging from- emotionally evocative information^7^. Dysregulation in the stimulus-driven system is theorized to drive initial orienting biases toward- and increased disruption of goal-directed behavior by- motivationally relevant, but task irrelevant, information.

### Internal attention (dys)regulation

*Internal attention* is the processing of information stored in the mind, recalled from long-term or active in working memory^4^. Emerging work indicates that the goal-directed/stimulus-driven systems which govern *external-perceptual* processing may also subserve processing of *internal* events (e.g., memories, thoughts)^4,10,11^. Executive control processes, such as working memory and response selection, are by definition *internal and goal-directed* processes^13^, whereas other forms of cognitive processes and states may be characterized as *internal and stimulus-driven* processes. For example, unwanted memories or involuntary remembering have been conceptualized as an automatic reflexive bottom-up process dependent on stimulus-driven forms of internal attention, or any interesting memory that enters consciousness and takes over attentional resources^14,15^. Similarly, spontaneous thought has been characterized as *undirected* and often without awareness of its ontogeny (see^16^). We propose that much like how goal-directed and stimulus-driven attentional systems may subserve external attention and its dysregulation over motivationally-relevant information^7,17^, goal-directed/stimulus-driven systems may also subserve internal attention and its dysregulation (e.g., difficulty disengaging from thoughts, attentional capture by unwanted memories, etc.).

### Internal attention dysregulation, cognitive vulnerability, and mental health

Internal attentional processes are theorized to be functionally important to mental health^6,18–21^. Specifically, dysregulated internal attentional processes may subserve a variety of higher-order cognitive processes which have long been implicated in anxiety and depression such as repetitive negative thinking (e.g., rumination, worry), emotional (dys)regulation, and self-focused attention^6,22–25^. It has been theorized, for example, that internal attention processes contribute to rumination and depression via the narrowing scope of attention to mood congruent thoughts^26^, impaired disengagement from self-referential information^18^ and subsequent difficulty inhibiting no-longer relevant information from working memory^27^.

Empirical, albeit indirect, evidence for these ideas may be found in working memory (WM) bias research. WM and attention are closely related processes^11,28–30^—both characterized by the selective allocation of limited-capacity processing resources between competing objects (information)^4^. Recent meta-analyses report that anxiety and worry are associated with reduced performance in attentionally-demanding (but emotionally-neutral) WM tasks^31^. Similar effects have been found for depression, although these effects are more likely observed when attention is not entirely constrained to the WM task, providing opportunity for spontaneous task-unrelated thoughts and negative repetitive thought to impair performance^32,33^. In addition, mounting evidence suggests that persons with elevated levels of repetitive negative thinking^22^ also show impaired inhibition or disengagement from no-longer relevant information in WM (independent of the emotional valence of the information)^34^. This body of work provides important albeit indirect evidence as to the specific or unique role(s) of internal attentional processes (e.g., biased selection, impaired disengagement) in cognitive vulnerability and mental health. Although strong and important effects, they provide only indirect evidence with respect to internal attention per se. Indeed, WM tasks by design also rely on memory processes (e.g., encoding, storage, proactive interference) affecting task performance and make inferences regarding the role(s) of internal attentional processes based on the cognitive operations required to complete these tasks^35,36^.

### Gaps in the study of (dys)regulated attention: theory and method

Despite the theorized importance of dysregulated internal attention for cognitive vulnerability and mental health problems^6,18,22,26,27^, decades of research focused on external attention and its dysregulation^7^. This may be, in large part, explained by key constraints of the methodological paradigm through which (external) attention has long been measured and quantified. To measure (external) attention, methods require the capacity to experimentally control the timing, the content (or features), and the location (for spatial attention) of stimuli^37–39^. Thus, unlike readily experimentally controlled external sensory-perceptual stimuli, similar experimental control over the timing and content of a person’s thoughts may be the stuff of science fiction. We thus have no direct behavioral or cognitive-experimental task designed to measure and quantify internal attention and its dysregulation^1,40^.

Instead, to-date, we have relied on tasks such as the Sternberg and n-back designed to measure working memory processes thought to rely upon or indirectly reflect internal attentional processing^41,42^. We argue that the field may benefit from the development of a novel experimental approach to more directly measure and quantify internal attentional processing and its dysregulation. We propose that one promising means to do so is to experimentally *manipulate the phenomenology* of what are, in fact, external stimuli so that they are functionally processed and experienced as if they were internal events (i.e., one’s own thoughts). In other words, the experimental study of internal attention could be significantly advanced through the capacity to deliver stimuli that are phenomenologically-valid simulations of experience such as one’s own thoughts.

### Simulated thoughts paradigm and studies overview

We therefore developed the Simulated Thoughts Paradigm (STP). The STP is designed to deliver idiographic stimuli that simulate the *content* and the *experience* of one’s own verbal thoughts. First, we select a unique set (see Method section) of emotionally negative self-referential sentences (e.g., "I'm so alone.") and emotionally neutral self-referential sentences (e.g., "I have class soon.") per participant. Second, we audio record participants saying these selected self-referential thoughts out loud. With these simulated thought stimuli, we are able to experimentally control the timing, idiographic content^1,43^, emotional valence and intensity of participants’ own thoughts. The premise and rationale for this approach is that the idiographically-relevant content, timbre and tempo of one’s own internal voice is designed to elicit a phenomenological sense of ownership, authorship and identification with the experimentally controlled simulated thought-like stimuli that parallels similar phenomenology of one’s own spontaneous thoughts^44–46^. Thus, the STP is designed to elicit an experience that *feels* like thinking one’s thoughts. These STP stimuli thus become phenomenologically-valid simulations of experiencing one’s thoughts. Consequently, the STP methodology is designed to phenomenologically trick brain source localization—to mis-experience (external) audio stimuli that we experimentally control, with their own internally-generated mental events.

Own-voice thought stimuli as a means to simulate verbal thought is grounded in work on behavioral and phenomenological accounts of inner speech^47,48^ and cognitive neuroscience of own-voice perception and self-representations^49,50^. Hearing one’s own voice activates right prefrontal brain regions implicated in abstract self-representations and self-referential processes (not activated for example when hearing others’ voices)^49,51,52^ nor when simply reading text^53^. Furthermore, our approach to simulated thought through one’s voice is also informed by *embodied* or *simulated* accounts of cognition^54^. Broadly, mental representations or cognition are internally generated through simulation (activation) of the same modality-specific brain systems that sub-serve the encoding of perception, action and interospection. Accordingly, verbal thought is internally generated through simulation (activation) of left inferior frontal gyrus language-processing systems thought to sub-serve language production and comprehension^55,56^. Thus, own-voice thought stimuli provide a powerful means to simulate verbal thought due to the shared neural substrate between internal thought—and external speech-production and comprehension.

**Figure 1:**
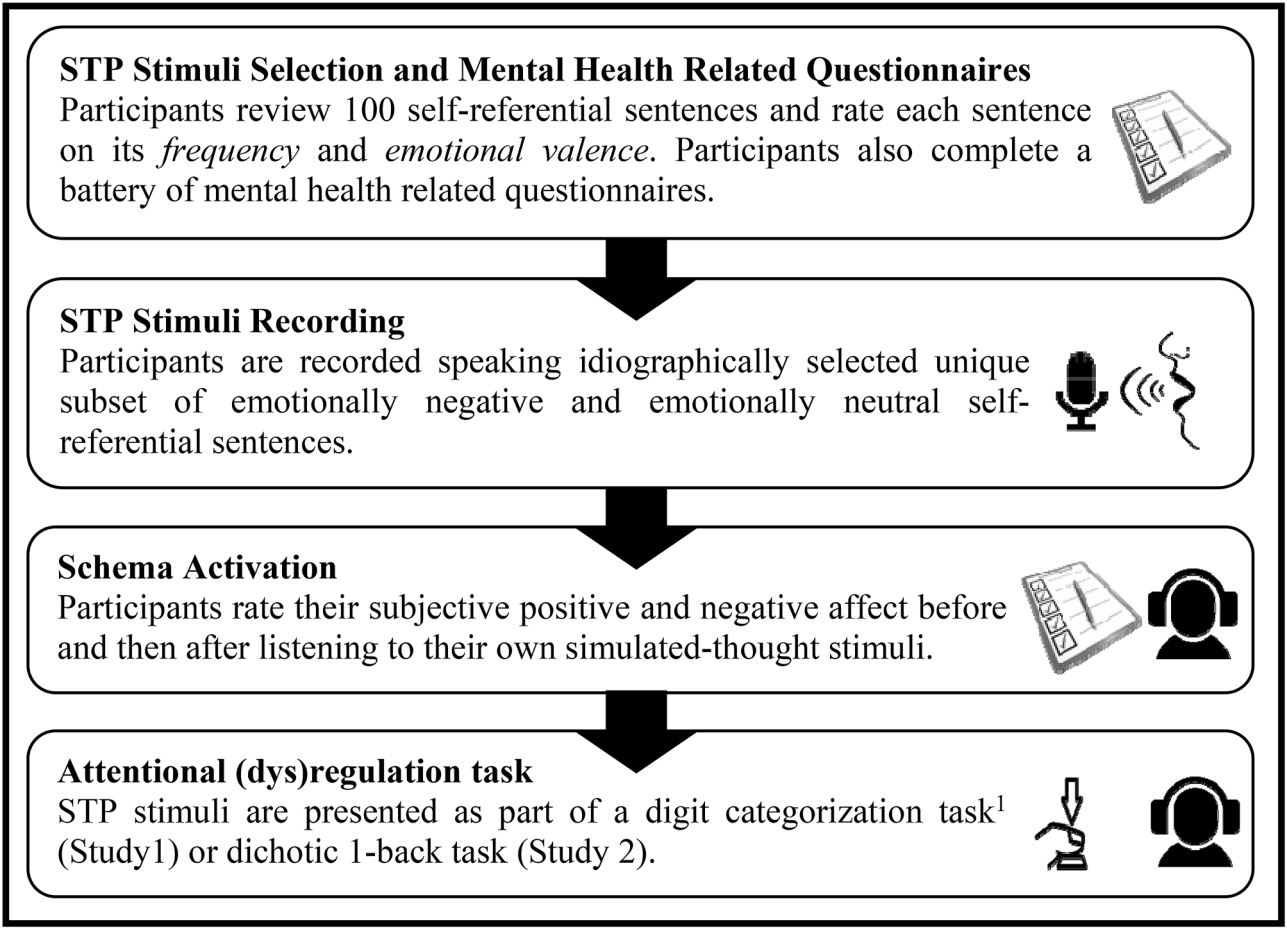
Experimental Procedure. STP = Simulated Thoughts Paradigm. ^1^Instructions and practice trials for the Digit Categorization Task were completed before the Schema Activation procedure.

**Figure 2:**
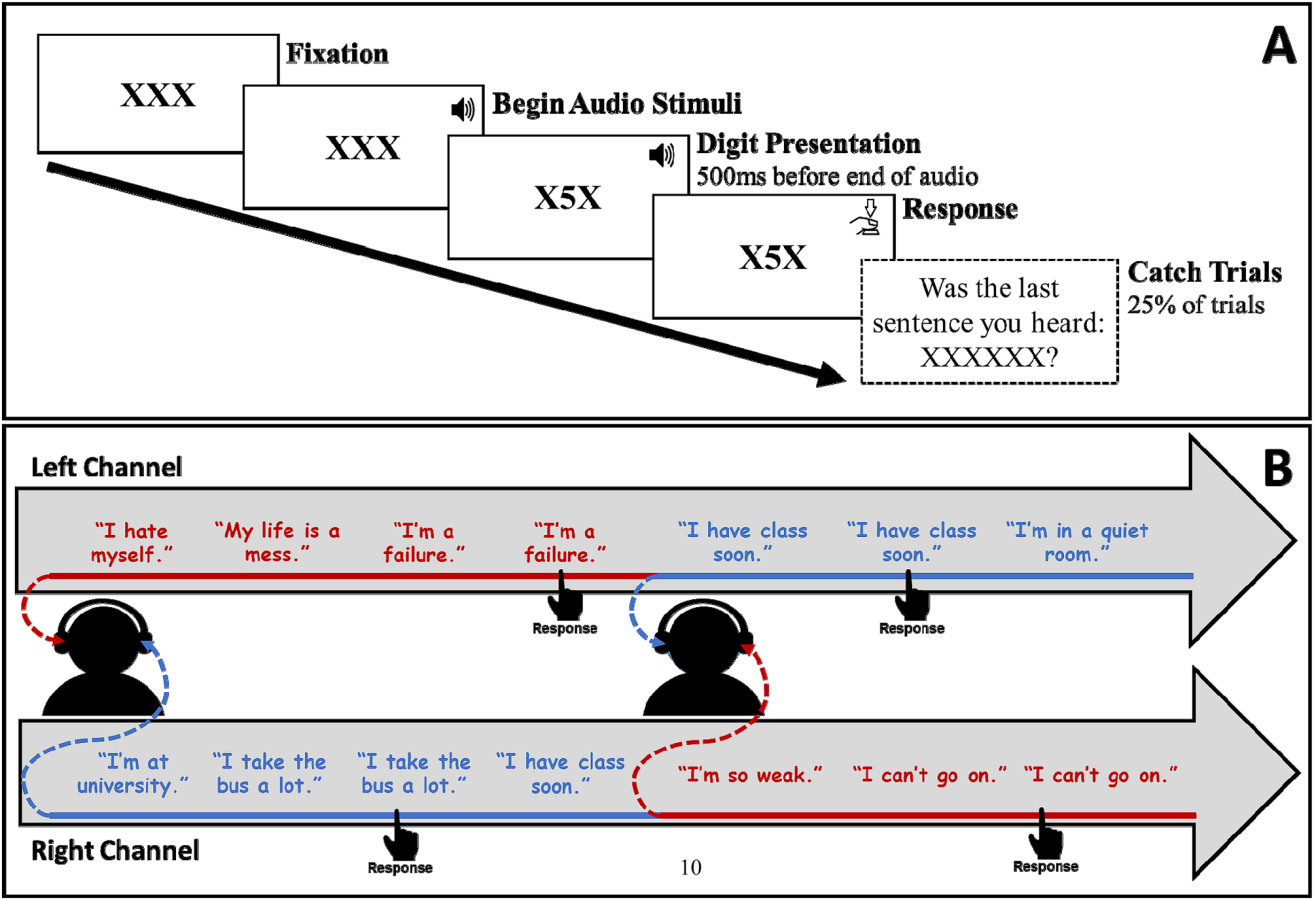
(**A**) Digit Categorization Task. Each trial begins with three Xs (horizontally aligned) presented at the center of the screen. After 1000 ms, participants hear an auditory negative self-referential or neutral self-referential sentence. Five-hundred ms before the end of the auditory stimulus the central X is replaced by a single visual target stimulus digit number (from 1 to 8) until response. Participants are instructed to press one of two keys categorizing the target digit as odd or even After response to the target digit participants were randomly (25% of trials) probed regarding the content of the sentence they heard during that trial. On 50% of catch-probe trials, the sentence presented (‘XXXXXX’ in the figure) was the same sentence heard, and on 50%, a different (incorrect) sentence was presented. Accuracy of catch trials: M(SD) = 99.46(1.99)%. (**B**) Dichotic 1-Back Task. Participants hear two separate lists of auditory stimuli, one list in each channel (ear). Stimuli lists are randomly mixed into intra-block sequences (i.e., 12 STP stimuli/sequence) of negative and neutral self-referential thoughts. When one channel (e.g., left side) delivers a negative stimulus the opposite channel (i.e., right side) delivers a neutral stimulus. At pseudo-random intervals, the simulated thought stimulus in one of the channels is presented sequentially (i.e., specific STP recording is repeated). Participants are asked to, as accurately and quickly as possible, press one of two buttons corresponding to the channel (LEFT/RIGHT) in which the stimulus was repeated sequentially. Biased selective internal attention is computed by subtracting accuracy in responding to repetitions in neutral stimuli from accuracy in negative stimuli. A positive bias score reflects greater selective attention to negative vs. concurrent neutral stimuli.

In Study 1, we used the STP to test and measure (1) subjective emotional reactivity to the experience of one’s own negative self-referential thoughts; and (2) internal attentional processing of those thoughts and, more specifically, difficulty disengaging internal attention from negative self-referential thoughts to external visual stimuli and task (visual digit categorization;^57^). First, we quantified emotion reactivity by measuring individual differences in degree of change in subjective ratings of positive and negative affect in response to the experience of one’s own self-referential thought (see Fig. 1 for experimental procedure) as well as activating participants’ negative self-referential schema via exposure to their negative self-referential simulated thought stimuli. Then, STP stimuli were presented as part of an established experimental task (digit categorization—odd or even; See Fig. 2A;^57^). By integrating STP into this adapted digit categorization task, we control the content and timing of both internal thought-like STP stimuli and external-perceptual task-response-relevant target. We thereby measure and quantify difficulty disengaging internal attention *from* simulated personalized negative self-referential thought (vs personalized emotionally-neutral self-referential thought) *to* task-relevant external information. Finally, prior to the lab session, participants completed a self-report battery including measures of negative repetitive thinking (repetitive negative thinking, brooding, worry)^58–60^ as well as depression and anxiety symptoms^61,62^. See Method section below for additional details. In Study 2 we ran a conceptual replication of Study 1 and similarly measured emotional reactivity as well as repetitive negative thinking, rumination, worry and depression and anxiety symptom levels. Critically, in Study 2 we examined the role of selective internal attention to negative (vs. neutral) self-referential thoughts (see Fig. 2B). To guard against p-hacking and inflation of family-wise alpha, all variables and analyses tested in studies 1 and 2 are reported in this manuscript; no additional measures or variables, beyond those reported here and in the Supplemental Materials (SM), were analyzed.

### Studies 1 and 2

#### Aims

**Figure 3:**
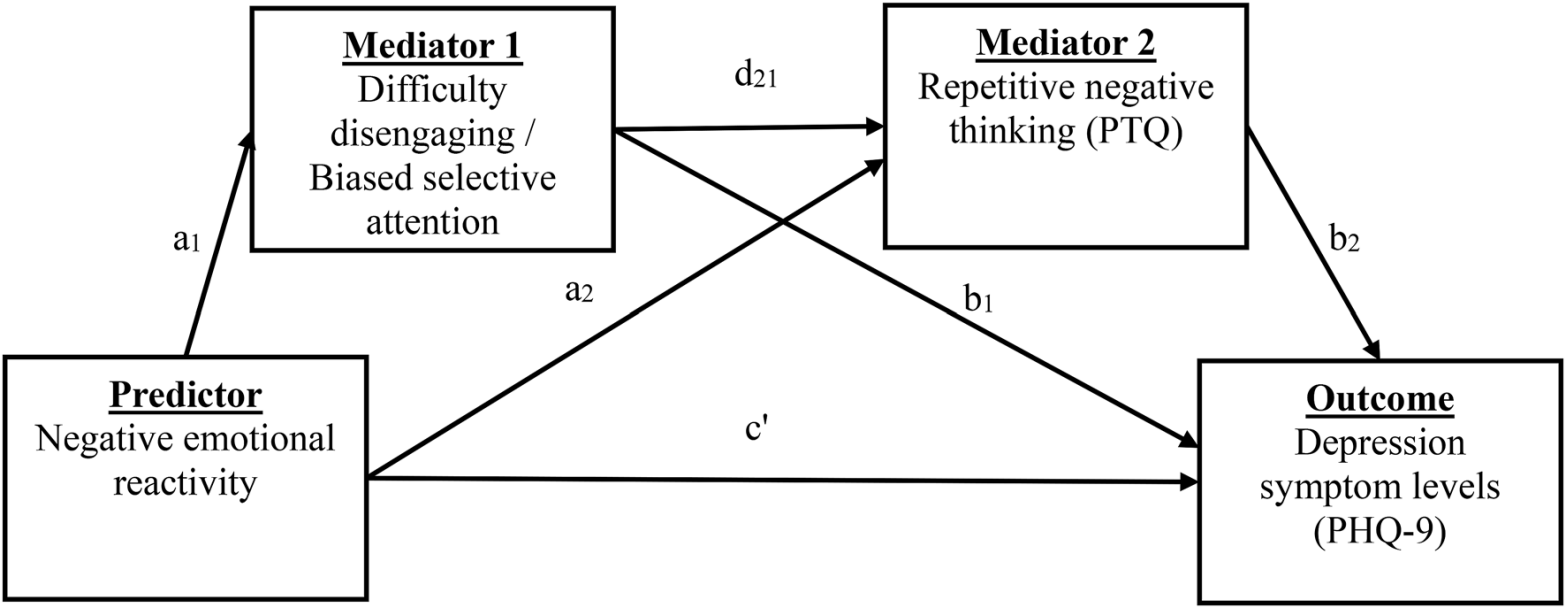
Diagram of serial mediation Model 1 in Study 1 and Study 2. Serial indirect effect of predictor on outcome through mediators 1 & 2 = *a*_1_**·d**_21_**·b**_2_. PHQ-9 = Public Health Questionnaire, PTQ = Perseverative Thinking Questionnaire. Study 1: *Interference scores* were entered as the first mediator (Mediator 1) to estimate difficulty disengaging internal attention from negative self-referential thoughts and Perseverative Thinking Questionnaire scores to estimate repetitive negative thinking as second mediator (Mediator 2). In this Model 1, depression symptom levels (PHQ-9) were entered as an outcome variable and anxiety symptom levels (Beck Anxiety Inventory) as an outcome in Model 2. In addition to these two primary serial mediation models, alternative mediator 1 and 2 and predictors were tested. First, to contrast the differential functional role(s) of negative and positive emotion reactivity, we tested a model wherein the predictor was change (reduced levels of) positive affect (emotion reactivity) to one’s negative self-referential thoughts as opposed to change in negative affect. Second, to examine the hypothesized specificity of attentional *interference* scores, we also tested the serial mediation models wherein dynamic *facilitation* scores as well as the *aggregated difference* scores were entered as mediator 1. Third, to examine the generalizability of the effects beyond a single construct and measure of cognitive vulnerability (i.e., repetitive negative thinking), we tested additional models wherein brooding or worry were entered as mediator 2. Study 2: Implemented the same design with *biased selective internal attentional score* as the first mediator.

Using the STP, we sought to study key predictions of central models of cognitive vulnerability of depression and anxiety implicating internal attention and its dysregulation in mental health^18,22,26,27,63^. In Study 1, we hypothesized that degree of emotional reactivity (i.e., elevated negative or reduced positive emotions) to experiencing (simulated) negative self-referential thoughts will predict degree of difficulty disengaging internal attention from these thoughts (vs. emotionally-neutral thoughts) to (emotionally-neutral) task-relevant external (visual) information^63,64^. Second, we hypothesized that degree of difficulty disengaging internal attention from negative self-referential thoughts will be associated with elevated levels of negative repetitive or perseverative thought, maladaptive rumination (brooding) and worry, as well as depression and anxiety symptoms^18,26,27^. Finally, per major models of cognitive vulnerability, we expected that these associations will function in a serial multiple mediation process. Emotional reactivity to negative self-referential thoughts will lead to difficulty disengaging from negative self-referential thoughts, this difficulty in disengaging will drive problems with negative repetitive self-referential thought processes (e.g., brooding), which will contribute to degree of depression and anxiety symptom levels^18,22,27,63^ (See Fig. 3). In Study 2, we aimed to replicate the predicted findings in Study 1. Specifically, we hypothesized that the same pattern of associations and serial mediation will be observed with respect to biased selective internal attention. Indeed, we expected emotion reactivity to negative self-referential thoughts would similarly contribute to biased selective attention to negative information and, in turn, drive cognitive vulnerability and thereby symptoms^32,65–67^.

## Results

### Study 1

#### Data scoring and analytic plan

**Figure 4:**
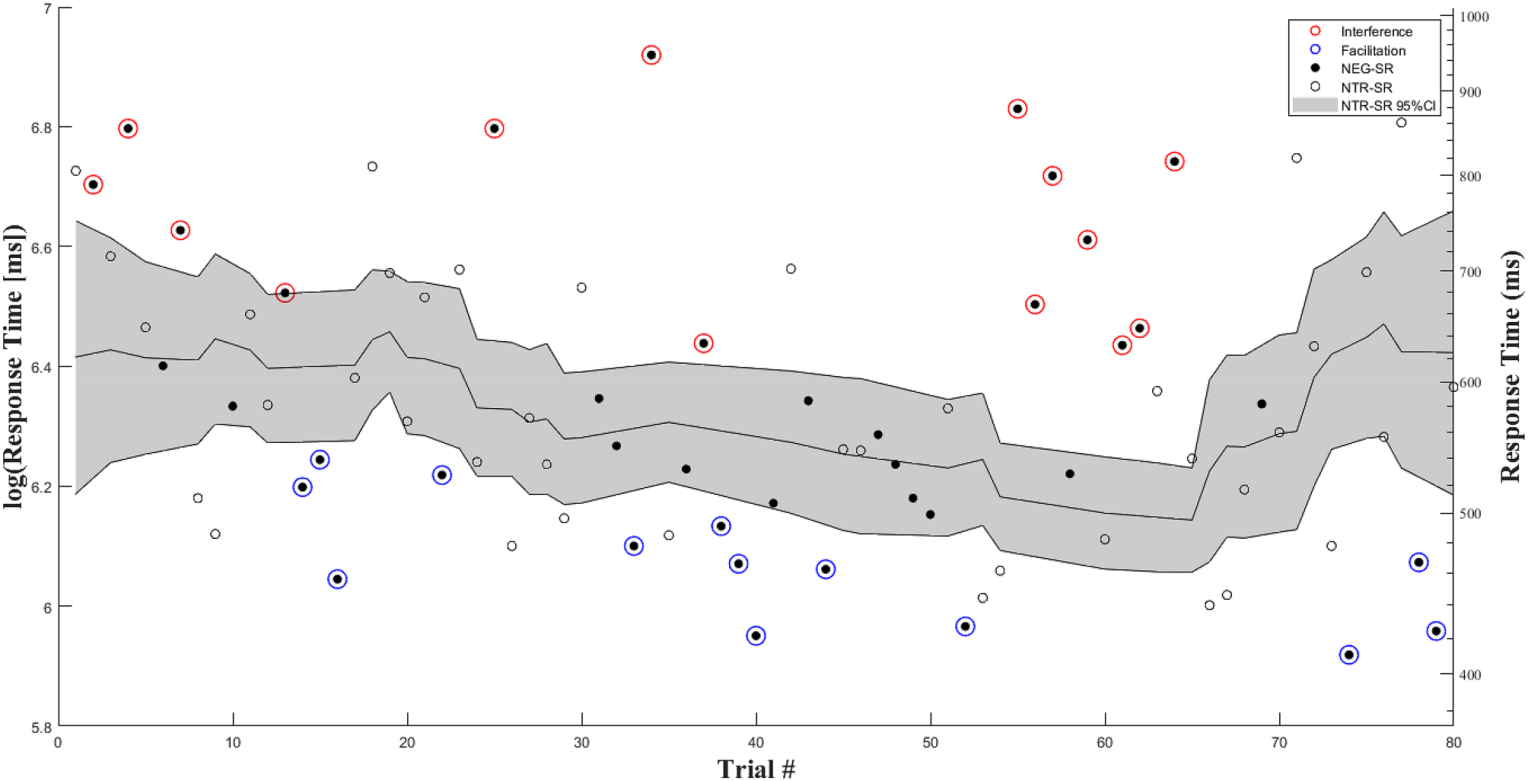
Example of response times across trials of the Digit Categorization Task. Black dots reflect RT on negative trials relative to white dots which reflect RT on emotionally neutral trials. Red circles around dots reflect trials slower than the upper-bound (+ 1.96SEs) of confidence of interval based on the RTs of a running window of 9 emotionally neutral trials (gray area). Blue circles around dots reflect trials faster than the lower-bound (-1.96SEs) of that running confidence of interval. Interference/facilitation scores are calculated by taking the mean of absolute difference scores (RT of each negative trial minus the running mean of neutral trials at that point in time [black line in gray area]) of interference/facilitation trials and dividing by that window’s running 1 SD. Aggregated mean bias scores are calculated by subtracting mean RT of all neutral self-referential trials (white dots) from mean RT of all negative self-referential trials (black dots).

We computed a positive emotional reactivity score and a negative emotional reactivity score from the subjective emotional state ratings reported by participants at baseline and then immediately following the schema activation wherein participants listened to their personalized neutral and negative self-referential stimuli (see Method section). For the digit categorization task we calculated three scores reflecting attentional disengagement from simulated thought stimuli. (1) A time-sensitive dynamic *interference score* (i.e., difficulty or delayed disengagement of internal attention from negative self-referential thoughts) and (2) a dynamic *facilitation score* (i.e., facilitated or faster disengagement of internal attention from negative self-referential thoughts). Both dynamic scores are based on mean of difference score (RT of each negative trial minus the running mean of neutral trials) of trials which are *faster/slower* than the upper/lower-bounds (± 1.96 Standard Errors [*SE*s]) of a confidence interval of the mean of a running window of 9 neutral self-referential trials and divided by that window’s running 1 standard deviation (SD) to correct for variability in neutral responses in time (see Fig. 4 and Method for additional details). (3) An *aggregated mean difference score* (i.e., the overall delayed [> 0] or facilitated [< 0] disengagement from negative relative to neutral self-referential trials) by subtracting mean RT of neutral self-referential trials from mean RT of negative self-referential trials.

We tested study aims and predictions by examining specific paths within serial mediation models (see Fig. 3). Serial multiple mediator models^68^ were estimated with bias-corrected and accelerated (BCa) bootstrapped confidence intervals^69,70^. To aid in interpretation of individual paths within the serial mediation model we also report zero-order Pearson correlations where possible (see also SM Table 2). All significance tests are two-tailed.

#### Digit categorization task: reliability

The observed split-half reliability (based on the average of 1000 random split halves of the task)^71^ was acceptable for the (1) dynamic *interference* (*r*(48) = 0.637, 95%CI 0.631–0.643) and (2) *facilitation* (*r*(48) = 0.524, 95%CI 0.516–0.532) scores, but low for the (3) *aggregated difference* score (*r*(48) = 0.291, 95%CI  0.282–0.301). Scores are Spearman-Brown prophecy corrected.

#### Association between emotional reactivity to- and difficulty disengaging internal attention from- negative self-referential thoughts

We first tested the hypothesized association between emotional reactivity to one’s negative self-referential thought and difficulty disengaging internal attention from those thoughts to an external visual task. To do so, we examined the association between the predictor (negative or positive) emotional reactivity scores and first mediator (M1) attentional interference, and separately (as alternate M1s) dynamic facilitation and aggregated bias scores scores—i.e., path *a*_1_ in the serial mediation model (see Fig. 3). As predicted, negative emotional reactivity scores were associated with interference scores (*Coeff* = 0.472, *BootSE* = *0.172*, *BCa95%CI* = 0.050 to 0.745) (*r*(48) = 0.447, *p* = 0.001) but not facilitation scores (*Coeff* = 0.023, *BootSE* = 0.048, *BCa95%CI* − 0.063 to 0.125) (*r*(48) = 0.067, *p* = 0.650). Likewise, negative emotional reactivity scores were associated with aggregated difference scores (*Coeff* = 16.398, *BootSE* = 6.234, *BCa95%CI* 3.832 to 29.014) (*r*(48) = 0.309, *p* = 0.033). In contrast to negative emotional reactivity, positive emotional reactivity scores were not significantly associated with interference scores (under bias-corrected and accelerated bootstrapped confidence intervals; *Coeff* = −0.244, *BootSE* = *0.152*, *BCa95%CI*  0.081 to − 0.513) (*r*(48) = −0.304, *p* = 0.035), facilitation scores (*Coeff* = −0.053, *BootSE* = 0.046, *BCa95%CI* = −0.153 to 0.031) (*r*(48) = 0.204, *p* = 0.165), nor aggregated difference scores (*Coeff* = −3.845, *BootSE* = 5.436, *BCa95%CI* = −18.247 to 3.231) (*r*(48) = −0.095, *p* = 0.519).

#### Association between difficulty disengaging internal attention from negative self-referential thoughts and cognitive vulnerability

**Figure 5:**
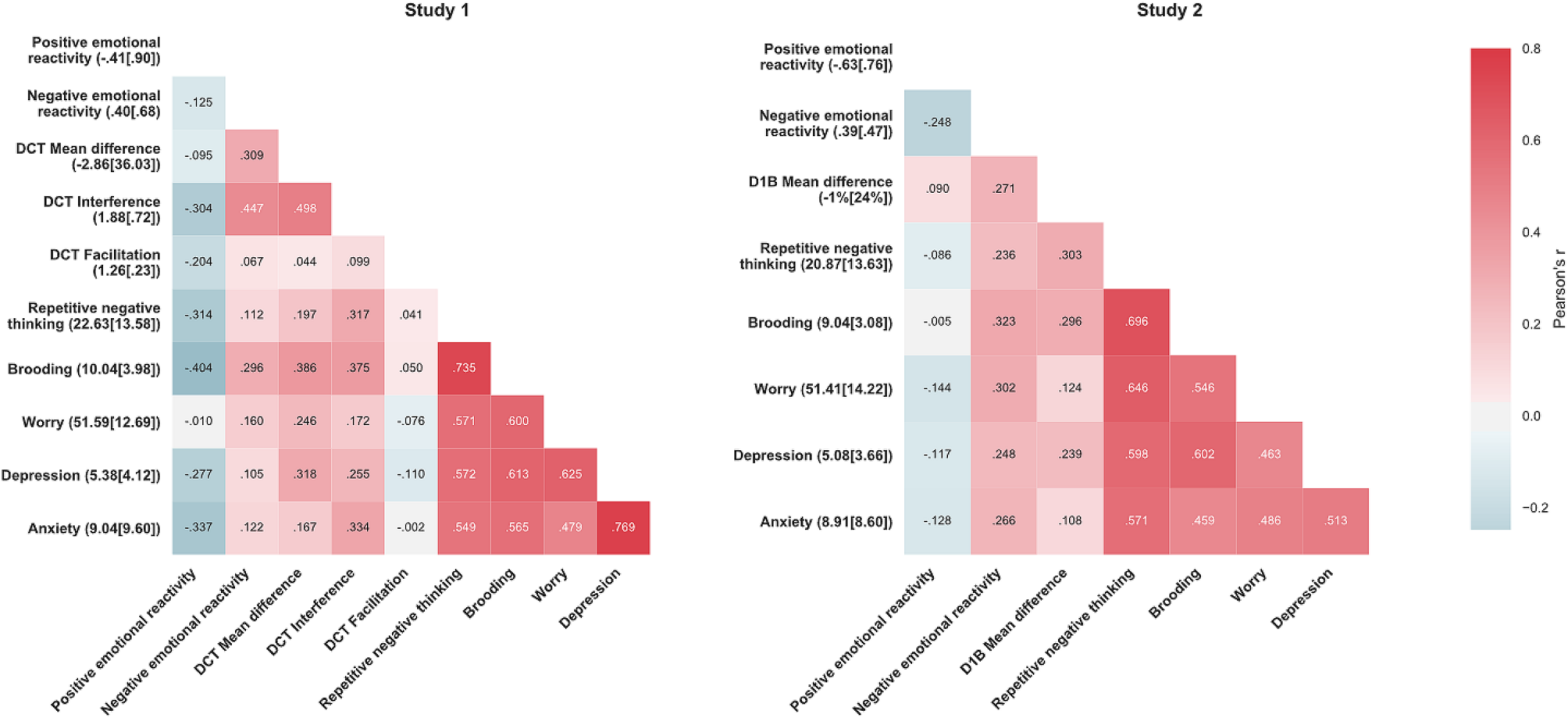
Heatmaps of zero-order Pearson correlations and descriptive statistics of variables (Mean [Standard Deviation]) in y-axis labels of Studies 1 and 2. DCT = Digit categorization task, D1B = Dichotic 1-back.

We next tested the hypothesized association between difficulty disengaging internal attention from negative self-referential thoughts and problems with negative repetitive thinking. We examined the association between M1 attentional interference, and separately dynamic facilitation and aggregated difference scores (alternate M1s), and second mediator (M2) repetitive negative thinking, and separately brooding, worry (alternate M2s)—i.e., path *d*_21_. Negative emotional reactivity was the predictor in the model. As predicted, internal attentional interference scores were significantly associated with repetitive negative thinking (*Coeff* = 6.330, *BootSE* = 2.940, *BCa95%CI* = 0.775–12.393) (*r*(48) = 0.317, *p* = 0.028). Similar significant associations were observed for brooding (*Coeff* = 1.687, *BootSE* = 0.833, *BCa95%CI* = 0.073–3.385) (*r*(48) = 0.375, *p* = 0.009) but not worry (see SM). Moreover, all models with worry as the second mediator showed non-significant associations in path *d*_21_ as well indirect effects (path *a*_1_*d*_21_*b*_2_; see below). Thus, for brevity, we do not further report on worry here (but see Fig. 5 and SM for details). Additionally, we tested the same associations when positive emotional reactivity was the predictor and attentional interference as M1—no significant associations were observed (see SM for details).

As predicted, facilitation scores were not associated with repetitive negative thinking (*Coeff* = 1.983, *BootSE* = 9.507, *BCa95%CI* = 25.413 to − 13.910) (*r*(48) = 0.041, *p* = 0.780) nor brooding (*Coeff* = 0.512, *BootSE* = 2.362, *BCa95%CI* = − 4.742 to 4.681) (*r*(48) = 0.050, *p* = 0.738). Aggregated difference scores were also associated with repetitive negative thinking (*Coeff* = 0.068, *BootSE* = 0.046, *BCa95%CI* = 0.008–0.196) (*r*(48) = 0.197, *p* = 0.180) as well as brooding (*Coeff* = 0.036, *BootSE* = 0.013, *BCa95%CI* = 0.011–0.062) (*r*(48) = 0.386, *p* = 0.007).

#### Association between difficulty disengaging internal attention from negative self-referential thoughts and depression and anxiety symptoms

We next tested the hypothesized association between difficulty disengaging internal attention from negative self-referential thoughts and depression and anxiety symptoms. To do so we examined the association between M1 attentional interference, and separately dynamic facilitation and aggregated bias scores (alternate M1s), and the outcome depression and anxiety—i.e., path *b*_1_ (see Fig. 3). Inconsistent with prediction, there was no direct significant association between attentional interference scores and depression (*Coeff* = 0.445, *BootSE* = 0.711, *BCa95%CI* = −0.856 to 1.977) (*r*(48) = 0.255, *p* = 0.081) or anxiety symptoms (*Coeff* = 2.483, *BootSE* = 2.107, *BCa95%CI* = −1.042 to 7.452) (*r*(48) = 0.334, *p* = 0.020). Furthermore, no direct associations were observed between attentional facilitation or aggregated mean difference scores and depression or anxiety.

#### Serial mediation of emotional reactivity and depression and anxiety by difficulty disengaging attention from negative thoughts and negative repetitive thinking

Finally, we tested the hypothesized serial multiple mediating mechanism linking negative emotional reactivity, difficulty disengaging internal attention, repetitive negative thinking, and symptom outcomes. Accordingly, we examined the serial indirect paths of the estimated models—i.e., the cross product of paths *a*_1_, *b*_2_ and *d*_21_. See Fig. 3. Results indicated a significant positive serial indirect path of M1 attentional interference and M2 repetitive negative thinking mediating the relations between negative emotional reactivity and depression (*Effect* = 0.495, *BootSE* = 0.356, *BCa95%CI* 0.060–1.660). A similar pattern of indirect effects or evidence for serial mediation was observed with respect to anxiety symptoms (*Effect* = 1.040, *BootSE* = 0.828, *BCa95%CI* 0.121–3.881). Notably, similar serial indirect effects were observed when brooding (rumination) was tested as M2, with outcomes of depression (*Effect* = 0.511, *BootSE* = 0.389, *BCa95%CI* 0.058–1.819) and anxiety (*Effect* = 1.017, *BootSE* = 0.883, *BCa95%CI* 0.092–4.490). No significant serial indirect effects were observed in models wherein positive emotional reactivity was the predictor (see SM for details). Furthermore, consistent with observed evidence of specificity of interference scores, we found no evidence of serial indirect effects for the dynamic attentional facilitation scores for either depression (*Effect* = 0.008, *BootSE* = 0.079, *BCa95%CI* − 0.090 to 0.292) or anxiety (*Effect* = 0.018, *BootSE* = 0.168, *BCa95%CI*: − 0.216 to 0.599). Finally, when aggregated mean difference scores were entered as M1, similar effects to those observed for dynamic interference scores were observed, including significant serial mediation paths for repetitive negative thinking and brooding (M2) for depression and anxiety outcomes (see SM for details).

#### Sensitivity analysis: ruling out alternative accounts of observed effects

We ran additional analyses to examine the possibility that observed results were accounted for by some unexplained general slowing of RT, some artifactual RT variability, or a general neurocognitive dysfunction affecting the ability to sustain attention over time reflected in RT. In these data, such an artifact or confound, for example, could generate instability or variability of RT that is *unrelated* to differential internal attentional processing of the negative vs. neutral stimuli in the digit categorization task^72,73^. Accordingly, we computed *pseudo-interference scores*, replacing the RT from the negative self-referential trials in the computation with RT from the neutral self-referential trials^73^. Accordingly, for these pseudo-score analyses, participants’ scores reflect their mean RT of *neutral* self-referential trials which are *slower/longer* than the upper-bound (+ 1.96 *SE*) of a confidence interval of the mean of an interpolated running window of 9 neutral self-referential trials and divided by that neutral window’s running 1 SD. If effects are due to variability unrelated to attentional processing of negative self-referential thought content per se, then the pseudo-scores would have the same likelihood of catching an effect as a score based on the experimental data. Accordingly, we examined the serial mediation models reported for dynamic interference scores and traditional aggregated scores but with the pseudo-interference scores as the first mediator (M1). In both of these pseudo-interference score models, there is no evidence of serial indirect paths for depression (*Effect* = −0.003; 95%CI: −0.254 to 0.030) or anxiety (*Effect* = −0.008.; 95%CI: − 0.254 to 0.030) nor a trend for such an effect. These findings indicate that observed serial multiple mediation model effects reported above are not likely accounted for by some artifactual or confounding source of RT slowing or variability or a general neurocognitive dysfunction affecting the ability to sustain attention over time that may be related to cognitive vulnerability or depression/anxiety. Thus, as theorized, the observed serial mediation model effects are more likely accounted for by difficulty disengaging internal attention from negative self-referential simulated thought stimuli in order to attend to an external stimulus and task.

### Study 2

#### Power analysis

Monte Carlo simulated power analysis^74^, based on the correlation matrix from Study 1, determined that 75 participants were needed to replicate serial mediation findings with 80% power.

#### Data scoring and analytic plan

Positive and negative emotional reactivity scores were computed identically to Study 1—subtracting the mean positive/negative emotion ratings after schema activation from base-line subjective emotion ratings upon arrival to the lab. For the dichotic 1-back task, we calculated a *biased selective attention score* by subtracting the accuracy for neutral stimuli from the accuracy for negative stimuli. To simplify interpretation, accuracy difference scores are presented in percentages (i.e. from − 100 to + 100% accuracy difference). An accurate response (for each stimulus) was the correct left/right button press for the according left/right channel in which a repetition occurred—i.e., the stimuli delivered at trial *n−*1 was repeated on trial *n* (in the same channel); or the correct withholding of a response in the absence of sequential stimulus repetition (see Fig. 2B). Identical to Study 1, we tested study aims and predictions by examining specific paths within serial mediation models.

#### Dichotic 1-back task: reliability

Split-block reliability was estimated by calculating biased selective attention score for each of the three dichotic 1-back task blocks and averaging the correlations between blocks. The observed split-block reliability of the accuracy scores for neutral (*r*(74) = 0.927, *95%CI* 0.917–0.940) and negative self-referential stimuli (*r*(74) = 0.930, *95%CI* 0.925–0.935) conditions—such that individual differences in accuracy, by stimuli type, were highly reliable across blocks^71,75^. As is often the case^75,76^, the split-block reliability of the difference between the conditions was low (*r*(74) = 0.123, *Range* = −0.144 to 0.400).

#### Similarity and identification with STP stimuli

An important assumption underlying the STP framework broadly, and the use of own-voice personalized thoughts to simulate/manipulate internal source localization more specifically, is embedded in the degree to which participants’ are indeed more likely to identify with simulated thoughts which subjectively “feel” similar to their own internal dialogue/thoughts. Among 57 participants in Study 2, we tested whether participants’ subjective report of the similarity (“To what degree were the sentences you heard similar to thoughts that pass through your mind?”) of STP stimuli significantly associated with the degree to which they identified (“To what degree did you feel that you identified with the sentences you heard?”) with those simulated thoughts. As predicted, the more subjectively similar STP own-voice stimuli were to each participant’s own internal dialogue/thoughts, the more likely they were to identify with the STP stimuli (*r*(57) = 0.816, *p* < 0.001) (see SM for procedure details and SM Fig. 1 for scatter plot).

#### Association between emotional reactivity- and biased selective attention- to negative self-referential thoughts

Utilizing the mediation framework from Study 1, we first tested the hypothesized association between emotional reactivity to one’s negative self-referential thoughts and biased selective attention to negative self-referential thoughts. We examined the association between the predictor (negative or positive) emotional reactivity scores and M1 biased selective attention—i.e., path *a*_1_ in the serial mediation model (see Fig. 3). As in Study 1 and as predicted, negative emotional reactivity scores were associated with biased attention (*Coeff* = 1.207, *BootSE* = *0.422*, *BCa95%CI* = 0.371–2.035) (*r*(74) = 0.271, *p* = 0.020). Positive emotional reactivity scores were not associated with biased attention (*Coeff* = -0.002, *BootSE* = 0.004, *BCa95%CI* = 0.004 to − 0.011) (*r*(74) = 0.090, *p* = 0.445).

#### Association between biased selective attention to negative self-referential thoughts and cognitive vulnerability

We next tested the association between biased selective attention and problems with repetitive negative thoughts. We examined the association between M1 biased attention and M2 mediator repetitive negative thinking, and separately brooding and worry (alternate M2s)—i.e., path *d*_21_. Negative emotional reactivity was the predictor in the model. Consistent with Study 1 and as predicted, biased selective internal attention scores were significantly associated with repetitive negative thinking (*Coeff* = 1.477, *BootSE* = 0.682, *BCa95%CI* = 0.049 to 2.721) (*r*(74) = 0.303, *p* = 0.009). Also, consistent with Study 1, worry was not associated with biased selective attention (see SM). Moreover, all models with worry as the second mediator showed non-significant associations in path *d*_21_ as well indirect effects (path *a*_1_*d*_21_*b*_2_; see below). Thus, for brevity, we do not further report on worry here (but see Fig. 5 and SM for details).

Inconsistent with Study 1, brooding was not associated with biased selective attention when negative emotion reactivity was the predictor in the serial mediation model (*Coeff* = 0.290, *BootSE* = 0.1640, *BCa95%CI* = −0.061 to 0.583) (*r*(74) = 0.296, *p* = 0.011). Indeed, Study 2 zero-order Pearson correlations between biased selective attention and brooding were significant and consistent with Study 1. The null *d*_21_ path between biased selective internal and brooding (relative to repetitive negative thinking) in the serial mediation model was likely due to shared explained variance between negative emotional reactivity and biased selective internal attention with brooding^68^. Indeed, when positive emotion reactivity was the predictor in the same serial mediation model, we found significant associations between biased selective attention and repetitive negative thinking (*Coeff* = 171.416, *BootSE* = 63.475, *BCa95%CI* = 39.006–293.543) and brooding (*Coeff* = 0.385, *BootSE* = 0.147, *BCa95%CI* = 0.074–0.661), and again, not worry (*Coeff* = 0.824, *BootSE* = 0.729, *BCa95%CI* = −0.685 to 2.171).

#### Association between biased selective attention to negative self-referential thoughts and depression and anxiety symptoms

Similar to Study 1 and inconsistent with prediction, there was no direct significant association between biased selective internal attention scores and depression or anxiety symptoms (see SM) with either positive or negative emotional reactivity as predictor.

#### Serial mediation of emotional reactivity and depression and anxiety by biased selective attention to negative thoughts and negative repetitive thinking

Finally, we tested a serial mediation path identical to Study 1, except that biased selective internal attention was included in the model as M1 rather than the difficulty disengaging attention from negative self-referential thoughts. Below we report findings for serial mediation per tested form of cognitive vulnerability (M2). First, as in Study 1 and as predicted, we observed a significant positive serial indirect path of M1 biased selective attention and M2 repetitive negative thinking mediating the relations between negative emotional reactivity and depression (*Effect* = 0.268, *BootSE* = 0.179, *BCa95%CI*: 0.037 to 0.831). Likewise, serial mediation was observed with respect to anxiety symptoms (*Effect* = 0.636, *BootSE* = 0.464, *BCa95%CI*: 0.087 to 2.181). Second, as in Study 1 and as predicted, for brooding as M2 we observed significant serial indirect effects when anxiety was the outcome (*Effect* = 0.421, *BootSE* = 0.303, *BCa95%CI*: 0.003 to 1.299) although not for depression (*Effect* = 0.237, *BootSE* = 0.161, *BCa95%CI*: − 0.005 to 0.654). Finally, as in Study 1 and as expected, no significant serial indirect effects were observed in models wherein positive emotional reactivity was the predictor (see SM for details).

#### Sensitivity analysis: ruling out alternative accounts of observed effects

As in Study 1, we ran additional analyses to rule out that observed serial mediation results were accounted for by individual differences in task performance or accuracy due to a general neurocognitive dysfunction affecting the ability to divide attention between dichotic channels rather than the theorized differential internal attentional processing of negative vs. neutral stimuli^32,67^. To rule out such a possibility, we replaced the biased selective attention mediator (M1) with an overall accuracy score across negative and neutral conditions. We found no evidence of serial mediation for depression (*Effect* = 0.013; 95%CI: − 0.016 to 0.350) or anxiety (*Effect* = −0.008.; 95%CI: − 0.254 to 0.030) nor any associations between negative emotional reactivity (predictor) and overall accuracy (M1) or between overall accuracy and any form of cognitive vulnerability (i.e., repetitive negative thinking, brooding, worry) (M2). Reported serial mediation effects thus are not likely accounted for by a general neurocognitive dysfunction.

## Discussion

The study of internal attention and its dysregulation has evaded direct cognitive-experimental study for decades. We thus developed the Simulated Thoughts Paradigm (STP)-to generate and deliver phenomenologically-valid thought-like stimuli; and thereby to experimentally study and measure internal attentional processing of these stimulated thoughts. In independent experiments, we thus sought to study the mediating role of internal attention and its dysregulation in linking “low-level” processes of emotional reactivity to the experience of one’s own negative self-referential thoughts and “higher-level” processes of cognitive vulnerability and related anxiety and depression symptomatology. In Study 1 (N = 48) we examined the role of difficulty disengaging internal attention from negative self-referential thoughts^18,23,26,27,77^. In Study 2 (N = 74) we examined the role of selective internal attention to negative self-referential thoughts^32,65,67^.

First, we found that the greater the negative emotional reactivity in response to negative self-referential thoughts, the greater the difficulty to disengage internal attention from those thoughts (Study 1) and the greater selective attention was biased to those thoughts (Study 2)^19,23,64,78,79^. Second, we found that difficulty disengaging (Study 1) from, as well as internal biased selective attention (Study 2) to, negative self-referential thoughts were associated with self-reported levels of repetitive negative thinking and brooding (rumination)^18,58^. Finally, we found evidence for a serial multiple mediation process in which (a) negative emotional reactivity to negative self-referential thoughts predicts (b) internal attentional dysregulation in the form of difficulty disengaging from (Study 1), as well as biased selective internal attention to (Study 2), negative self-referential thoughts; both forms of internal attentional dyscontrol predict (c) multiple forms of cognitive vulnerability (e.g., brooding); which, in turn, predict (d) degree of depression and anxiety symptom severity. These effects appear to be robust, observed in two independent samples (N = 122) across two key forms of internal attentional processing and unique respective cognitive-experimental tasks (digit categorization and dichotic 1-back) using the STP to deliver simulated thought stimuli.

These serial mediation effects provide novel and rigorous support for major theories implicating emotional reactivity to negative experiences and states, attentional processing and control, and cognitive vulnerability to depression and anxiety^18,26,27,67^. Broadly, common to these theories is the idea that maladaptive thinking processes (e.g., repetitive negative thinking, brooding) are maintained and exacerbated by a self-sustaining cycle of negative emotion(s) that triggers internal content (e.g., negative self-referential thoughts) which potentiate further negative emotions and so forth^80^. In turn, rumination (brooding or similar processes) exacerbate increase probability of initial symptoms turn into episodes of major depression or anxiety (see^66^). These cognitive vulnerability theories differ on the specific implicated cognitive process that fails to adaptively regulate the cognition-emotion cycle—such as difficulty disengaging attention from self-referential information^18^ or dysfunction in inhibiting (removing) negative contents in WM^27^. These findings, especially in Study 1, provide initial and to-date the most direct evidence in support of the disengagement hypothesis^18^. Notably, we did not find direct associations between difficulty disengaging attention from- nor biased selective attention to- negative self-referential thoughts and depression or anxiety symptomatology. Effects of internal attentional dysregulation in Studies 1 and 2 on anxiety and depression were observed only when mediated via cognitive vulnerability. Consistent with attentional dysregulation and cognitive inhibition accounts of depression and anxiety^18,22,26,27,34^, this may mean that the effects of these dysregulated “lower-level” internal attentional processes are directly linked “higher-level” cognitive vulnerability processes and only thereby to symptomatology. This effect may have important implications for further specifying major models of information processing and cognitive vulnerability^6,65^; and, in turn, translational implications for delimiting outcomes and mechanisms of action of training interventions targeting “lower-level” internal attentional dyscontrol^81^.

Observed effects in Study 1 were specific to difficulty disengaging internal attention as theorized^18,19^. Indeed, no effects were observed at the zero-order level or within the serial mediation model for attentional facilitation (i.e., facilitated internal attentional disengagement from simulated negative self-referential thought stimuli). The specificity of these effects provides strong evidence for cognitive control theories implicating attentional dyscontrol or difficulty disengaging internal attention in cognitive vulnerability^18,27^. Moreover, this approach to disambiguate interference or difficulty disengaging attention from facilitated disengagement is consistent with emerging efforts to develop time-sensitive dynamic indices of attentional processing using cognitive-experimental task data^17,72,73,82–85^. Indeed, the approach utilized here—contrasting RT on individual target (emotion) trials relative to a running window of comparator (neutral) trials that simultaneously corrects for degree of variability in RT that is unrelated to attentional processing of the target trial (simulated thought) stimulus—may represent a useful advance in the study and quantification of biased or dysregulated attentional processing of emotional information that is sensitive to dynamic changes in mean and variability of RT across time. It is also noteworthy that we found similar effects when difficulty disengaging internal attention was quantified through the traditional aggregated difference mean bias score measure. Consistent with recent criticism and debate regarding the utility and limitations of such traditional aggregated difference scores to estimate biased attentional processing^73,84,85^, we found that the traditional aggregated mean bias score demonstrated significant albeit weaker zero-order effects relative to the dynamic interference scores as well as relatively weaker levels of internal reliability relative to the dynamic scores^71,84,85^. These weaker effects may reflect the confounding or admixture of trials on which a participant expressed facilitation with trials in which she/he expressed interference.

Relatedly, it is important to note that the dynamic interference and facilitation scores were based on a computation sensitive to RT slowing and variability reflected in (a) within-subject dynamic changes in time (i.e., trials) as well as (b) between-subject differences in RT variability. This was done by, repeatedly, taking the difference between (i) the RT on each negative self-referential target trial and (ii) the running mean RT of that target trial’s reference running window of neutral trials and (iii) dividing this trial-level difference by the SD of that reference running window of neutral trials. Accordingly, the within-subject correction for neutral trial RT variability should account for any within- and between-subject individual differences effects driven by artifactual or confounding slowing and related variability of RT not specifically related to slowed disengagement from target trial stimulus content (i.e., simulated negative self-referential thought stimulus). Moreover, we observed strong evidence of specificity for the dynamic interference scores (cf. facilitation scores) across all zero-order and mediation model analyses. If artifactual or confounding RT variability was the sole or primary source of observed effects, then we would not find the observed specificity for dynamic interference (cf. facilitation scores) scores with respect to correlates and outcome of interest. Indeed, under such conditions, both interference and facilitation dynamic scores would have the similar/identical likelihood of association with measured correlates and outcomes in these data, as both sets of dynamic scores would be a byproduct of artifactual RT variability. Likewise, the reliability and replicability of Study 1 effects are strengthened by Study 2 findings. Indeed, Study 2 scoring of biased internal attentional processing entailed direct trial-level accuracy computations and thus cannot be accounted for by some computational artifact.

Moreover, sensitivity analyses in Study 1 and 2 ruled-out that a general neurocognitive dysfunction or general difficulty sustaining attention over time linked to cognitive vulnerability or depression/anxiety account for observed effects^86^. Likewise, sensitivity analyses in Study 1 ruled-out that general slowing or intra-subject variability of RT over time due to measurement error or some other unexplained source of variability, unrelated to problems with internal attentional disengagement from self-referential negative thoughts, account for observed effects.

One unexpected and noteworthy effect was observed. In contrast to negative repetitive thought and brooding, worry was not associated with difficulty disengaging internal attention from- nor biased selective attention to- negative self-referential thoughts. These findings are contrary to findings from studies documenting WM impairment in depression and anxiety wherein rumination and worry are similarly associated to an underlying deficit in cognitive control^34^ and emerging perspectives that rumination and worry are functionally similar processes^22^ (but see^87^). We speculate that the avoidance theory of worry^88^ may help account for these differential effects. Unlike negative repetitive thought or brooding, worry may not be primarily driven by internal attentional dysregulation but by a history of negative reinforcement contingencies^6^. That is, it may be that attentional dysregulation of negative self-referential thoughts may not maintain worry in anxious individuals. Instead, worry effectively diverts attention away from unpleasant experiences (internal imagery and/or external somatic experiences) in the short-term, and is thereby negatively reinforced and thus maintained and amplified^88^. If replicated, this finding may have theoretical implications for cognitive models of worry and anxiety^6^; and, in turn, translational implications for delineating the potential therapeutic utility of training internal attentional (dys)control (i.e., indicated for brooding but not worry). An alternative methodological account of these differential effects is that we simulated negative self-referential thoughts. We did not simulate feared anxiety provoking-thoughts about unwanted future events. Were we to have done so, perhaps we would have observed similar dysregulated internal attention from those simulated future-oriented feared thought stimuli and worry, and in turn, anxiety (see SM Discussion for further unexpected finding related to brooding).

These studies may have a number of implications for basic and clinical science. First, the STP is a novel methodology for experimental control and manipulation of simulated thought-like stimuli. It is unique in its capacity to simulate not only the *content* of personal thoughts but the *experience* of thinking those thoughts in a controlled experimental context^49^. In addition to facilitating the capacity to experimentally study internal attentional processing of (simulated) thoughts, it is more broadly an example of a paradigm to deliver *stimuli that are phenomenologically-valid internal experience(s)*^46^. This was by design in contrast to the more typical stimuli that represent external events or information in our environment most often the focus of our cognitive-experimental research^4,81^. This approach is in step with growing interest in recent years in the study of internal experiences, such as mindwandering, meditation and self-generated thought, and the cognitive (e.g., internal attention) and neural bases (e.g., default-mode network) subserving these mental phenomena^89–91^. Interest in such phenomena has driven various forms of measurement method innovations such as experiential sampling and daily diaries^1,92^, random probing and self-caught report as well as behavioral tasks designed to capture off-task thinking^43,92^, and brain-imaging of spontaneous thoughts^64,91^ (see^93^ for a review). STP adds paradigmatically, by offering the capacity to not only observe but to experimentally control/manipulate the content- and timing of- verbal thought-like experiences. This may have a number of applications. For example, the STP may be relevant to experimental study of theories and models of psychopathology that emphasize the nature and function of thoughts and thought content^94,95^. Such applications may, for example, include intrusive memories in trauma^96^, biased (negative) interpretations in social anxiety^97^ or thought obsessive–compulsive disorders^98^.

Moreover, these findings may have implications for understanding what specific cognitive processes—e.g., internal attention, short-term memory—may specifically subserve cognitive vulnerabilities such as brooding and repetitive negative thinking. In Study 1, there were no (or at least extremely limited) demands for memory. In each trial, participants simply need to attend to the simulated internal (thought-like) stimuli and then disengage and engage with the external visual target. A single STP stimuli item is not expected to tax working-memory capacity^36^. As such, finding an association between difficulty disengaging attention from negative thoughts and brooding and cognitive vulnerability suggests that these internal attentional processes, and not inter-related working memory processes, account for cognitive vulnerabilities. These findings provide novel empirical support to the disengagement hypothesis and attentional scope theories^18,26^ relative to theories implicating broader working-memory dysfunctions^27^. However, future work could more directly test this relation. For example, this may be done by delivering two separate tasks measuring both attentional and memory processes; or, by developing novel experimental task that concurrently manipulates both attentional and memory processes^35^. Such study may more directly delineate the unique role of lower-level attentional and mnemonic processes in higher-level cognitive vulnerabilities.

Finally, findings may also have implications for experimental therapeutics. Could training internal attentional control or capacity to selectively attend to or disengage from one’s thoughts lead to reduced cognitive vulnerability and thereby improved mental health^18^? Likewise, do interventions that effectively engage change in “higher-order” processes in cognitive vulnerability do so via change in internal attentional (dys)control^99^? For example, mindfulness-based intervention models hypothesize that meditation practice, in part, targets internal attention by training voluntary selection, orienting and disengagement from internal objects of experience (e.g., mind-wandering) and thereby salutary and curative effects of mindfulness training (e.g., via greater cognitive flexibility and reduced reactivity;^90,99^). Accordingly, the STP may provide a useful means to measure and monitor change in the capacity to regulate attention to private thoughts, in response to mindfulness training as well as other therapies that may similarly target attention and cognitive control processes^6,100^. Furthermore, the ability to measure the moment-to-moment dynamic expression of internal attentional dyscontrol may provide a unique training capacity—such as to deliver real-time feedback on internal attentional dyscontrol or momentary mental events characterized by biased selective attention to and difficulty disengaging internal attention from one’s thoughts^101,102^. Such technology may facilitate self-regulatory processes such as meta-awareness of attention to one’s (repetitive negative) thoughts and thereby internal attentional control.

The present studies have a number of limitations. First, the samples were limited to a university community sample, although the distribution of observed scores (central tendency, variability) of studied variables (e.g., rumination, worry, depression, anxiety) were representative of population norms^59,60,62^ (see SM Table 1). Thus, while findings provide initial insight into dysregulated internal attention in vulnerability to depression and anxiety, it may be important that future work also test these methods and findings among selected and clinical populations. Second, experimental stimuli types were limited to *only* self-referential, either neutral or negatively valenced, simulated thought stimuli and presented in participant’s own voice. Accordingly, we cannot rule out: (1) to what extent effects are independently driven by self-referentialitiy vs. emotional valence (negative vs. neutral)? (2) Likewise, we do not know whether the same stimuli content, presented in another person’s voice, may potentially elicit similar interference effects? The selected stimuli and their delivery were designed to provide optimal conditions to simulate the phenomenology of thoughts^47,48^—important for the initial proof-of-principle test of the paradigm and theory. Indeed, thought stimuli were designed to reflect thought content central to theories of cognitive vulnerability in depression^18,27,66^. The use of one’s voice was grounded in behavioral and phenomenological accounts of inner speech, cognitive neuroscience of own-voice perception and self-representations, and embodied cognition accounts of verbal thought^47,49,51,54^. Future research could, for example, be designed to dissociate internal attentional processing of self-referential thought content from motivationally-salient content or negative- from positive-valenced thought content, or between modes for audio delivery of simulated thought stimuli. In addition, participants were instructed to listen to STP stimuli “as if they were thoughts passing through their mind”. However, as in hundreds of other studies^103,104^, we were not able to readily examine whether or to what degree participants were able to apply this mental stance and attitude towards the stimuli. Future work could explore the possibility of a manipulation check that helps to quantify the degree to which participants apply or attempt to apply the prescribed mental instructional set and whether doing so is relevant for study outcomes. Furthermore, beyond brief subjective phenomenological interviews which we conducted with participants, we do not have strong empirical basis to know how well the STP *simulates* the phenomenological experience of verbal thinking. Likewise, we are only simulating the *experience* of verbal thought. We thus do not know the extent STP makes these simulated thought stimuli *feel* like they are “internal”. As is the case in most experimental work, readers should keep in mind the gap between the theoretical construct of interest (internal attention) and operational-methodological approach (STP) for studying this construct. Nevertheless, we believe that this is a first, and we hope, useful step towards more direct experimental study of internal attentional processing of thoughts. Finally, qualitative methods may permit unique insight into the perceptual-experiential experiences STP mimics and/or triggers. For example, neurophenomenological methods^105^ or micro-phenomenological interviews^106^ maybe useful in better understanding the degree to which STP stimuli elicit phenomenological sense of ownership, authorship, identification or agency over simulated thoughts.

In summary, we propose that (dys)regulation of internal attention may be fundamentally important to (mal)adaption and mental health. Accordingly, we introduced a novel paradigm to experimentally simulate the content and experience of thought (STP). We found that difficulty disengaging from and biased selective attention to negative self-referential thoughts explains relations between negative emotional reactivity to one’s thoughts, problems with perseverative negative thinking, and, in turn, depression and anxiety. Study findings provide novel evidence for models implicating internal attentional (dys)control in cognitive vulnerability for depression and anxiety.

## Method: study 1

### Participants

Forty-eight participants were recruited from a university community in Israel (*M(SD)*_*age*_ = 24.46(6.11) years-old, range_age_ 18–39; 74% female). Potential participants were excluded from the study if they were: (a) < 18 years-old; (b) report hearing or speech difficulties; or (c) mother tongue other than Hebrew (to reflect their native internal language-of-thought), not fluent in Hebrew, or report thinking in a language other than Hebrew ("In what language do you typically think? For example, when recalling a phone number."). All participants provided informed written consent prior to participation. Experimental procedures were approved by the Department of Psychology Ethics Committee (University of Haifa) and followed the relevant ethical guidelines regulations.

### Measures and apparatus

#### Simulated thought paradigm (STP): stimulus selection

The STP methodology is designed to deliver idiographic stimuli that simulate the *content* as well as the *experience* of one’s own verbal thoughts. Briefly, participants reviewed a list of 100 self-referential verbal thought-like sentence items, of which 67 items were derived from established questionnaires (see SM) and classified apriori as negatively valenced (e.g., "I'm so alone.") and 33 as emotionally neutral (e.g., "I have class soon."). Participants rated (1) how *frequently* they have thoughts similar to each item, on a 5-point scale (1 = "Never have this thought" to 5 = "Think about it a lot";^95^) and (2) to what *degree* the item/thought elicits negative or positive emotion on 7-point scale (*valence level*; -3 = "Very negative", 0 = "Neither positive nor negative", 3 = "Very positive."). For each participant, we selected the 20 negative self-referential items with highest frequency (*M(SD)* = 2.114(0.892)) and negative valence (*M(SD)* = −2.262(0.740)) ratings, and 20 neutral self-referential items with highest frequency (*M(SD)* = 2.450(1.126)) and nearest to neutral valence (i.e., smallest absolute value; *M(SD)* = 0.078(1.126)) ratings (i.e., ratings nearest to 0). Participants were then recorded speaking each sentence aloud, and these recordings where used as simulated thought stimuli within the Schema Activation and Digit Categorization tasks (below). STP methodology included modification of simulated thought stimuli using a low-pass filter. This was done to more closely simulate the perceptual sonics of hearing one’s own voice—an approach that has been previously demonstrated to increase subjective ratings of the likeness of these voice stimuli to hearing one’s own voice^49,107^. We observed a significant difference in mean duration of negative (*M(SD)* = 1.529 (0.348) seconds) and neutral (*M(SD)* = 1.456 (0.286) seconds) stimuli in a paired samples t-test (*t(47)* = 5.052, *p* < 0.001). See SM for analysis ruling out duration difference scores in explaining the observed effects. See SM for additional details of specific instructions, equipment and software used for the STP recording and delivery.

#### Simulated thought paradigm (STP): schema activation

Participants were instructed to listen to the simulated thought stimuli “as if they were thoughts passing through your mind”. Participants reported their subjective positive and negative affect (Subjective Emotional State; see Questionnaires below) immediately after listening to the sentences (Time 1) as well as when initially arriving at the lab (Time 0). Negative emotional reactivity (NER) was calculated from subjective state emotion ratings by subtracting participants mean ratings of *negative* emotions (guilt, anxiety, anger, sadness, embarrassment, distress) before and after listening to their own simulated-thought stimuli (Time 1—Time 0). Scores > 0 reflect elevation in negative emotion following exposure to their simulated thoughts. Positive emotional reactivity (PER) was similarly computed based on mean rating of *positive* emotions (interest, joy, amusement) before and after listening to their own simulated-thought stimuli (Time 1—Time 0). Scores < 0 reflect reduction in positive emotion in response to the simulated thoughts.

#### Simulated Thought Paradigm: Digit Categorization Task (DCT)

See Fig. 2. STP stimuli were presented via an established experimental task augmented to measure and quantify difficulty disengaging internal attention *from* simulated personalized negative self-referential thought (vs personalized emotionally-neutral self-referential thought) *to* task-relevant external information. Specifically, the DCT was designed to measure attentional disengagement from stimulated thoughts (negative and neutral self-referential) stimuli to a digit categorization (odd or even) task^57^. The task consisted of 80 trials with trial type determined by the auditory stimulus (40 negative self-referential trials, 40 neutral self-referential trials; randomly presented/alternated from trial-to-trial). During each trial, three Xs (horizontally aligned) were presented at the center of the screen. After 1000 ms, participants heard an auditory negative self-referential or neutral self-referential sentence. Five-hundred ms before the end of the *auditory* stimulus the central X was replaced by a single *visual* target stimulus digit number (from 1 to 8) until response. Participants were instructed to press one of two keys categorizing the target digit as odd or even. Dependent measure was RT, reflecting time to disengage from (negative and neutral self-referential) stimuli and respond to digit target. Catch trials were used to ensure that participants processed the content of each and every simulated thought stimulus; and to motivate participants to attend to the auditory negative/neutral self-referential stimuli in a goal-directed manner so that we could help ensure that RT to external visual digit stimuli reflect speed of internal attentional disengagement from the simulated thought stimuli. Specifically, randomly following 25% of trials and immediately following participants’ response to the digit, one of the 40 negative/neutral self-referential sentences was presented on the monitor. On these catch trials, participants were instructed to indicate whether the presented sentence was or was not the last simulated thought. On 50% of these catch trials the correct answer was YES. Accuracy on catch trials was high (*M(SD)* = 99.46(1.99)%) indicating that participants indeed attended to simulated thought stimuli.

##### Digit categorization task (DCT): mean aggregated difference scores

The mean RT of neutral self-referential trials were subtracted from the mean RT of negative self-referential trials, such that a greater positive score reflects slower disengagement or greater interference aggregated across negative self-referential trials, and greater negative score reflects faster disengagement or greater facilitation.

##### Digit categorization task (DCT): dynamic scores

The above noted *aggregated difference score* represents the traditional computation from similar cognitive-experimental tasks of external attention^17,40^. It has, however, also been the subject of extensive criticism and debate in recent years^73,84,85^. Accordingly, we computed two dynamic scores in line with recent efforts to develop time-sensitive dynamic indices of attentional processing using cognitive-experimental task data^17,73,82–85^. *Attentional interference.* A running mean (9 trials window: current trial (n) and 4 trials forwards (n + 4) and 4 trials backwards(n−4)) and 95% confidence interval (95%CI; RTs are log transformed for normality prior to calculation of CI) were calculated for neutral trials, and then linearly interpolated across the rest of time points (i.e., negative self-referential trials) that occurred between neutral self-referential trials. In other words, a mean and 95%CI of neutral trials RT is modelled in time for negative self-referential trials, such that each negative trial has both its "actual" RT (participant’s response time on that trial) and an estimation of RT and 95%CI of that estimation were, at that point in the task, a neutral self-referential occurred. We then (1) identify negative self-referential trials whose RT is greater—i.e., *slower*—than the 95%CI of neutral self-referential trials of that respective time-point; then (2) compute a trial-level difference score by subtracting the neutral running mean RT from that (negative) target trial's RT; finally, (3) divide that difference score by one standard deviation of neutral trials within the time window. A mean average score (of steps 2 and 3) is then calculated for all identified negative self-referential trial RTs. See Fig. 4. *Attentional facilitation.* Like the dynamic interference score, a similar procedure was used to compute attentional facilitation. For facilitation, however, mean RT time is calculated only from negative self-referential trials *faster* than their respective neutral CI, and the trial-level difference score is calculated by subtracting the negative self-referential trial RT from the neutral trials running mean RT. Magnitude scores thus provide an estimation of interference and facilitation on negative self-referential trials whose RTs are likely (95% confidence) outside of the distribution of neutral trials at that specific moment in the task.

### Questionnaires

Self-report questionnaires included mental health-related measures of repetitive negative thinking (Perseverative Thinking Questionnaire (PTQ)^58^), brooding—a maladaptive form of rumination (Rumination Response Scale (RRS)^60,108^), worry (Penn State Worry Questionnaire (PSWQ)^59^), as well as symptom levels of depression (Public Health Questionnaire-9 (PHQ9)^62^), and anxiety (Beck Anxiety Inventory (BAI)^61^) as well as momentary subjective positive and negative affect ratings (Subjective Emotional States (SES)^109^) for negative and positive emotional reactivity.

### Procedure

Prior to the lab session, participants completed an on-line survey in which they provided initial consent and completed a battery of questionnaires—including the stimuli selection process of the STP. In the lab, participants again provided consent for the experimental lab session, rated their baseline subjective state negative and positive affect (SES), and then completed the STP stimuli recording (see SM). Participants began the practice phase (8 trials of STP stimuli) of the DCT. Following these practice trials, and before continuing to the 80 trials of the DCT, we delivered the Schema Activation via the STP—participants passively listened to their own simulated thought stimuli and again rated their negative and positive subjective state affect (SES). After completing the experiment, participants were compensated.

## Method: study 2

### Participants

Seventy-four participants were recruited from a university community in Israel (*M(SD)*_*age*_ = 25.24(4.63) years-old, range_age_ 18–43; 70% female). Exclusion criteria was similar to Study 1 with the addition that individuals did not participate in the previous study. All participants provided informed written consent prior to participation. Experimental procedures were approved by the Department of Psychology Ethics Committee (University of Haifa) and followed the relevant ethical guidelines regulations.

### Measures and apparatus

#### Simulated thought paradigm (STP): stimulus selection

See Study 1.

#### Simulated thought paradigm (STP): schema activation

Schema Activation procedure was identical to Study 1 with one difference. Participants heard the 20 neutral STP stimuli and then immediately reported their subjective positive and negative affect immediately (Time 0), and then continue to hear the 20 negative STP stimuli and again reported their positive and negative affect ratings (Time 1). Negative/positive emotional reactivity (NER) was by subtracting participants mean ratings of negative/positive emotions at Time 0 from those at Time 1 (Time 1—Time 0). This was done so that we could further disentangle the effects of emotionally neutral from negative self-referential simulated thoughts on emotion reactivity observed in Study 1.

#### Simulated thought paradigm: dichotic 1-back task (D1B)

This task is a novel integration of the established Dichotic Listening and 1-back experimental paradigms^110^ designed to more closely reflect selective attention between concurrent thought-like stimuli. Participants heard two separate lists of STP stimuli, one list in each channel (ear). Stimuli lists were randomly mixed into intra-block sequences of 12 negative or neutral self-referential thoughts. When the one channel (e.g., left side) delivered a negative stimulus the opposite channel (i.e., right side) deliverred a neutral stimulus. At pseudo-random intervals the stimuli in one of the channels was presented twice sequentially (i.e., specific STP stimuli is repeated) and participants were instructed to, as accurately and quickly as possible, press one of two buttons corresponding to the channel (LEFT/RIGHT) in which the same stimuli was repeated sequentially (see Fig. 2).

More specifically, intra-block sequences consisted of ten unique STP stimuli of similar valence, with two stimuli randomly repeated, accordingly for a total of 12 stimuli per chunk. The rationale behind using intra-block sequences was to allow time for participants to recognize the content of stimuli in each channel and accordingly reorient their attention towards or away from that channel—potentially affecting their identification of a repetition (see below). For example, a participant may be attending to their left channel, where a neutral stimulus is presented. Participant may then reorient their attention to examine what stimulus is concurrently presented in the right channel. A negative stimulus in the right ear may capture, or maintain, their attentional focus on the right channel. Intra-block sequences and individual stimuli onset and offsets were not temporally matched between channels, meaning that (except for the very beginning of the task) stimuli in one channel may begin (once the previous stimuli in the same channel ended) while stimuli from the opposite channel is still on-going. The task consisted of 3 blocks with a brief self-timed rest period between blocks.

##### Dichotic 1-back task: mean accuracy difference scores

A *biased selective attention score* was calculated by subtracting the accuracy for neutral stimuli from the accuracy for negative stimuli. An accurate response (for each stimulus) was the correct left/right button press for the according left/right channel in which a repetition occurred—i.e., the stimuli delivered at trial *n−*1 was repeated on trial *n* (in the same channel); or the correct withholding of a response if no repetition was present (see Fig. 2B).

### Questionnaires

See Study 1.

### Procedure

The procedure was divided into 3 stages—one home stage and two lab sessions. Prior to the first lab session, participants completed an on-line survey in which they provided initial consent and completed a battery of questionnaires, as well as assessment necessary for idiographic STP stimuli selection. In the first lab session, participants provided consent for the experimental lab sessions, and then completed the STP stimuli recording stage. In the second lab session, participants first completed the Schema Activation via the STP—participants passively listened to their own simulated thought stimuli and rated their negative and positive subjective state affect (SES) first after hearing the neutral stimuli and again after hearing the negative stimuli. Participants then completed the Dichotic 1-Back Task practice and test task trials. After completing the experiment, participants were compensated.

## Supplementary information

Supplementary Information 1.
