# Supplementary material for: Simulating thoughts to measure and study internal attention in mental health
Source: Sci Rep. 2021 Jan 26;11:2251. doi: 10.1038/s41598-021-81756-w (PMC7838298; doi:10.1038/s41598-021-81756-w)
Supplement: Supplementary file 1 — Supplementary Information 1. [file 41598_2021_81756_MOESM1_ESM.docx]

**SUPPLEMENTARY MATERIALS**

Simulating Thoughts to Measure and Study Internal Attention in Mental Health

Iftach Amir, M.A.

Liad Ruimi, M.A.

Amit Bernstein, Ph.D.*

University of Haifa

*Corresponding author:

Prof. Amit Bernstein, Ph.D.

Director, Observing Minds Lab

Department of Psychology, University of Haifa

Mount Carmel, Haifa 31905, Israel

Email: [abernstein@psy.haifa.ac.il](mailto:abernstein@psy.haifa.ac.il)

Lab: http://irca-haifa-6h5e.squarespace.com/

Phone: +972-4-824-9659 | Fax: +972-4-824-0966

# SUPPLEMENTARY MATERIALS

# METHOD

## Simulated Thoughts Paradigm (STP)

Idiographic stimuli selection. A list of 67 NEG-SR was generated from items from three established questionnaires: The Beck Depression Inventory II (BDI-II;^1^) Rumination Response Scale (RRS; ^2^) and the Automatic Thoughts Questionnaire (ATQ; ^3^). Certain items from these questionnaires were rephrased to better mimic simple, self-referential, internal-dialogue-like phrasing. For example, the first RRS item "Think about how alone you feel." was changed to "I'm so alone." Additionally, overlapping content items between questionnaires ^2^ were removed (i.e., when two items had the same meaning, only one was used). We generated an additional 33 (neutral self-referential (NTR-SR) sentences, with sentences such as "I'm a university student." and "I'm participating in an experiment."

Stimuli Recording. Participants were recorded using microphones placed in the participants’ ears (Roland CS-10EM). This simple approach helps to better capture the sonic properties of hearing oneself when speaking relative to typical placement of microphones in-front of the mouth ^4^. First, participants were instructed to sit quietly, without moving, for 5 sec. During this 5 sec silent period background noise in the room was recorded (this recording is later used to filter background white noise from recorded stimuli). The participant was then instructed:

"You will now be asked to speak 40 sentences out loud, one sentence at a time. Each time you are asked to say aloud one sentence which will appear on the screen. For each sentence, **take a moment to say it to yourself in your head.** When you are ready, press the space bar, wait during the displayed 3 second countdown and then say the sentence out loud **as you pronounce it when speaking in your head.** When you finished speaking the sentence, press again the space bar."

The experimenter then guided the participant through three practice sentences. First, the twenty NTR-SR sentences were presented, followed by the twenty NEG-SR sentences. During the recording period, the experimenters listened "on-line" to the recording of each sentence. Sentences which were abnormal in fluency (e.g., include stuttering, coughing, participant forgets the sentence, etc.) were marked. Once the participant finished recording all forty STP stimuli, the experimental software repeated the procedure for the stimuli which were marked as abnormal – i.e., the participant then recorded those same stimuli once again. Finally, experimenters reviewed and edited recorded sentences, removing silent periods at the beginning and end of each recording, as well as removing background white-noise and then running a low-pass filter on the sound files. The low-pass filter was used because it was previously demonstrated to increase the subjective ratings own-voice familiarity (i.e., sounds like me) ^5^ due to the filter’s compensation for the loss of lower (bass) frequencies transmitted via skull-bone vibrations when hearing oneself while speaking. Moreover, we normalized maximum amplitude of recordings to -15dB to standardize simulated thought stimulus volume within- and between-subjects

## Questionnaires

In addition to scales reported in the manuscript, we also measured cognitive fusion or reactivity to thought content ^6^ as a related marker of cognitive vulnerability ^7^. Findings for cognitive fusion were nearly identical to those reported for other measured forms of cognitive vulnerability (brooding, repetitive negative thinking). For brevity, we thus report findings for cognitive fusion in the supplement only.

Cognitive Fusion Questionnaire (CFQ)^7^. The CFQ is 7-item self-report measure in which participants rate on a 7-point Likert-type scale (1 = "Never true" to 7 = "Always true") statements that reflect cognitive fusion – "the tendency for behavior to be overly regulated and influenced by cognition" ^7^. The CFQ has been conceptualized as a measure of degree of reactivity to thought content ^6^. The CFQ has demonstrated sound internal consistency and test-retest reliability ^7,8^.

STP Identification and Similarity ratings. In Study 2, participants were asked to report at the very end of the experiment, “To what degree were the sentences you heard similar to thoughts that pass through your mind?” (similarity) and “To what degree did you feel that you identified with the sentences you heard?” (identification). Both questions were rated a scale of 1 (“Not at all”) to 10 (“Very much”). We collected these data among N = 57 participants.

## Instruments

Software. On-line (home) questionnaire battery was delivered via Qualtrics. The lab procedure was administered via MATLAB, except behavioral tasks (Digit Categorization Task, Emotional reactivity) which were run in E-Prime 2 in Study 1. Audio was edited using Audacity 2.1.2 ^9^. ***Monitor.*** Displayed on Samsung S23A950D at 100hz. ***Participant Input.*** Participant responses in behavioral tasks were recorded using PST Serial Response Box. ***Audio.*** In-ear microphones: Roland CS-10EM; Headphone: Senheisser PC-350; Sound card: ASUS Xonar U7.

## Data preparation

Digit categorization task (DCT). Data was screened for inaccurate (*M(SD)* = 1.25(3.16)%) and outlier RTs 3 Standard Deviations (SD) from the mean of each participant's NEG-SR or NTR-SR conditions or RTs faster than 100ms (*M(SD)* = 1.77(1.09)%)^[[1]](#footnote-1)^.

## Data analytic plan

As describe in the main manuscript, we tested study aims and predictions by examining specific paths within the serial mediation models. Here we report statistical details of findings only summarized in the Results of the main manuscript. In addition to these primary serial mediation models reported in the main manuscript, we also report tested alternative M2 predictor of Cognitive Fusion Questionnaire as a measure of reactivity to one’s thought content.

# RESULTS

## Study 1

Association between difficulty disengaging internal attention from negative self-referential thoughts and cognitive vulnerability. We tested the hypothesized association between difficulty disengaging internal attention from negative self-referential thoughts and problems with negative repetitive thinking. We examined the association between M1 attentional interference, and separately dynamic facilitation and aggregated bias scores, and M2 mediator repetitive negative thinking, and separately brooding, worry – i.e., path *d*_21_; with negative emotional reactivity as predictor. As predicted, internal attentional interference score was significantly associated with repetitive negative thinking as well as brooding (see Main Manuscript); and cognitive fusion (*Coeff* = 5.837, *SE* = 2.103, *BCa95%CI =* .808 to 10.083) (*r*(48) = .391, *p* = .006). Facilitation scores were not associated with cognitive fusion (*Coeff* = 2.634, *SE* = 6.279, *BCa95%CI =* -6.530 to 18.878) (*r*(48) = .070, *p* = .639). Aggregated difference scores were not associated with cognitive fusion (*Coeff* = .048, *SE* = .042, *BCa95%CI =* -.018 to .141) (*r*(48) = .193, *p* = .189). Worry was not associated with interference scores (*Coeff* = 2.222, *SE* = 2.892, *BCa95%CI =* -2.342 to 7.941) (*r*(48) = .172, *p* = .242) nor were facilitation (*Coeff* = -4.755, *SE* = 8.490, *BCa95%CI =* -20.908 to 14.411) (*r*(48) = -.076, *p* = .608) and aggregated mean difference scores (*Coeff* = 2.222, *SE* = 2.892, *BCa95%CI =* -2.342 to 7.941) (*r*(48) = .246, *p* = .092).

We additionally examined the same paths (between models alternating repetitive negative thinking, brooding, worry and cognitive fusion as M1 and attentional interference as M2) but entered positive emotional reactivity as predictor. In all models *d*_21_ paths were nonsignificant. Internal attentional interference score was not associated with repetitive negative thinking (*Coeff* = 4.634, *SE* = 2.730, *BCa95%CI =* -.575 to 8.563), brooding (*Coeff* = 1.544, *SE* = .761, *BCa95%CI =* -.254 to 2.543); and cognitive fusion (*Coeff* = 4.240, *SE* = 1.901, *BCa95%CI =* -1.267 to 6.478) nor worry (*Coeff* = 3.299, *SE* = 2.726, *BCa95%CI =* -1.902 to 7.588).

Serial mediation of emotional reactivity and depression and anxiety by difficulty disengaging attention from negative thoughts and negative repetitive thinking. We tested the hypothesized serial multiple mediating mechanism linking negative emotional reactivity, difficulty disengaging internal attention, repetitive negative thinking, and symptom outcomes. Results indicated a significant serial indirect paths of M1 aggregated mean difference and M2 repetitive negative thinking mediating the relations between negative emotional reactivity and depression (*Effect* = .178, *BootSE* = .197, *BCa95%CI*: .026 to 1.173). A similar pattern of indirect effects or evidence for serial mediation was observed with respect to anxiety symptoms as outcome (*Effect* =.419, *BootSE* = .467, *BCa95%CI*: .046 to 2.745). Similar serial indirect effects were observed when brooding was tested as M2, with outcomes of depression (*Effect* = .366, *BootSE* = .282, *BCa95%CI*: .082 to 1.513) and anxiety (*Effect* = .849, *BootSE* = .659, *BCa95%CI*: .180 to 3.474) .

We additionally examined the serial indirect paths of M1 attentional interference and M2 cognitive fusion mediating the relations between negative emotional reactivity (as predictor) and depression and anxiety symptom levels (as outcome variables). Similar (see Main Manuscript) serial indirect effects were observed for cognitive fusion as second mediator (Depression as outcome: *Effect* = .767, *BootSE* = .463, *BCa95%CI*: .097 to 2.017; Anxiety as outcome: *Effect* = 1.616, *BootSE* = 1.071, *BCa95%CI*: .216 to 4.933) replacing repetitive negative thinking.

Additionally, we tested the serial multiple mediating mechanism linking positive emotional reactivity as the predictor. No significant serial indirect paths were observed for M1 attentional interference and M2 repetitive negative thinking mediating the relations between positive emotional reactivity and depression (*Effect* = -.179, *BootSE* = .178, *BCa95%CI*: -.802 to .008) nor with respect to anxiety symptoms (*Effect* = .365, *BootSE* = .381, *BCa95%CI*: -1.697 to .013). Similar serial indirect effects were observed when brooding (rumination) was tested as M2, with outcomes of depression (*Effect* = -.231, *BootSE* = .204, *BCa95%CI*: -.905 to .020) nor anxiety (*Effect* = -.432, *BootSE* = .426, *BCa95%CI*: -2.046 to .014).

Mean duration difference between negative and neutral stimuli as alternative explanatory variable. We scored mean duration difference by subtracting the mean duration of neutral from mean duration of negative stimuli per participant. The mean duration difference was not correlated with any measure except brooding (*r*(48) = .291, *p* = .045). We further examined whether this explained observed findings for brooding. We ran a multiple regression in which the relation of attentional interference with brooding remained significant (B = 2.101, p = .006) with mean duration as covariate (B = 12.300, p = .029). Furthermore, no serial indirect effect was observed for mean duration difference for depression (*Effect* = -.154, *BootSE* = .351, *BCa95%CI*: -1.227 to .290) nor anxiety (*Effect* = -.299, *BootSE* = .656, *BCa95%CI*: -2.586 to .363) as outcomes.

## Study 2

Association between biased selective attention to negative self-referential thoughts and cognitive vulnerability. We examined the association between M1 biased attention and alternate M2 mediator worry – i.e., path *d*_21_. Negative emotional reactivity was the predictor in the model. Biased selective internal attention did not predict worry levels (*Coeff* = .274, *SE* = .855, *BCa95%CI =* -1.693 to 1.718) (*r*(74) = .124, *p* = .292).

We additionally examined the serial models where *positive* emotional reactivity was the predictor (rather than negative emotional reactivity). In contrast to repetitive negative thinking and brooding (see main manuscript) worry was not associated with biased selective attention (*Coeff* = .824, *SE* = .729, *BCa95%CI =* -.685 to 2.171).

Association between biased selective attention to negative self-referential thoughts and depression and anxiety symptoms. Inconsistent with prediction, there was no direct significant association between biased selective internal attention scores and depression or anxiety symptoms with either positive or negative emotional reactivity as predictor: (a) negative emotional reactivity (NER) as predictor, repetitive negative thinking as M2 and depression as outcome: *Coeff* = .062, *SE* = .158, *BCa95%CI =* -.272 to .349; *r*(74) = .239, *p* = .040; (b) NER as predictor, repetitive negative thinking as M2 and anxiety as outcome: *Coeff* = -.386, *SE* = .354, *BCa95%CI =* -1.022 to .390; *r*(74) = .108, *p* = .358; (c) positive emotional reactivity (PER) as predictor, repetitive negative thinking as M2 and depression as outcome: *Coeff* = .132, *SE* = .149, *BCa95%CI =* -.181 to .399; (d) PER as predictor, repetitive negative thinking as M2 and anxiety as outcome: *Coeff* = -.248, *SE* = .331, *BCa95%CI =* -.834 to .495.

Serial mediation of emotional reactivity and depression and anxiety by biased selective attention to negative thoughts and negative repetitive thinking. As in Study 1, no significant serial indirect effects were observed in models wherein positive emotional reactivity was the predictor (see Table 2).

Consistent with Study 1 and contrary to prediction, non-significant indirect path was observed for worry as M2 with both depression (*Effect* = .036, *BootSE* = .121, *BCa95%CI*: -.234 to .274) and anxiety as outcomes (*Effect* = .089, *BootSE* = .329, *BCa95%CI*: -.802 to .620).

# DISCUSSION

Two effects were unexpected and may be noteworthy. First, within the serial mediation models, we did not observe significant associations between positive emotional reactivity (i.e., reduction in positive affect in response to listening to one’s self-referential thoughts) and difficulty disengaging attention from- (Study 1) or biased selective attention to- (Study 2) negative self-referential thoughts, nor evidence of serial mediation (see main manuscript). Such effects were, however, observed for negative emotion reactivity. Indeed, positive and negative reactivity are not related – degree of negative to one’s negative self-referential thoughts does not predict degree of reductions in positive emotion reactivity to those thoughts and vice-versa (see also ^10^). Accordingly, serial mediation findings indicate that positive and negative emotion in response to negative self-referential thoughts may thus serve distinct functions with respect “lower-order” (internal attentional disengagement) and “higher-order” cognitive vulnerability and, in turn, depression and anxiety ^11^. One *post-hoc* hypothesis of the difference between the null mediation model pathways relative to the significant zero-order associations is that variance in these processes linked to positive emotional reactivity is orthogonal from that accounted for by negative emotional reactivity. For example, whereas elevated negative emotion in response to threat or danger may function to capture attention ^12^, such as by narrowing the scope of (internal) attention to mood congruent information and related attentional dyscontrol processes ^13–15^ and thereby cognitive vulnerability and symptoms, reduced positive emotion may not serve the same function ^16^. Instead, positive emotion reactivity may be linked to cognitive vulnerability and symptomatology via processes that may unfold over longer periods of time such as behavioral withdrawal or autonomic inflexibility ^10,13^. Largely post-hoc speculation, this theorizing may represent a promising direction for further investigation of emotion, attention dyscontrol, and cognitive vulnerability in mental health. However, Study 2 correlations between positive emotional reactivity and brooding and repetitive negative thinking cast doubt over this post-hoc hypothesis. An additional unexpected finding of interest relates to an inconsistency between studies 1 and 2 in the association between dysregulated attention and brooding. In Study 1 we observed a significant positive association between difficulty disengaging internal attention from negative self-referential thoughts and brooding. In Study 2 we did not find a significant association between biased selective attention to negative thoughts and brooding. However, positive zero-order correlations were statistically significant in both studies. This suggests that, in so far as internal attentional processes implicated, difficulty disengaging attention from negative thoughts seems to be the main mediating process between reactivity and brooding.

# References

1. Beck, A. T., Steer, R. A. & Garbin, M. G. Psychometric properties of the Beck Depression Inventory. *Clin. Psychol. Rev.* **42**, 841–865 (1998).

2. Treynor, W., Gonzalez, R. & Nolen-Hoeksema, S. Rumination Reconsidered: A Psychometric Analysis. *Cognit. Ther. Res.* **27**, 247–259 (2003).

3. Hollon, S. D. & Kendall, P. C. Cognitive self-statements in depression: Development of an automatic thoughts questionnaire. *Cognit. Ther. Res.* **4**, 383–395 (1980).

4. Stenfelt, S. & Goode, R. L. Bone-Conducted Sound: Physiological and Clinical Aspects. *Otol. Neurotol.* **26**, (2005).

5. Shuster, L. I. & Durrant, J. D. Toward a better understanding of the perception of self-produced speech. *J. Commun. Disord.* **36**, 1–11 (2003).

6. Bernstein, A. *et al.* Decentering and Related Constructs: A Critical Review and Metacognitive Processes Model. *Perspect. Psychol. Sci.* **10**, 599–617 (2015).

7. Gillanders, D. T. *et al.* The Development and Initial Validation of the Cognitive Fusion Questionnaire. *Behav. Ther.* **45**, 83–101 (2014).

8. Hadash, Y., Plonsker, R., Vago, D. R. & Bernstein, A. Experiential self-referential and selfless processing in mindfulness and mental health: Conceptual model and implicit measurement methodology. *Psychol. Assess.* **28**, 856–869 (2016).

9. Audacity, T. Audacity®. (2017).

10. Tellegen, A., Watson, D. & Clark, L. A. On the Dimensional and Hierarchical Structure of Affect. *Psychol. Sci.* **10**, 297–303 (1999).

11. Watson, D., Wiese, D., Vaidya, J. & Tellegen, A. The two general activation systems of affect: Structural findings, evolutionary considerations, and psychobiological evidence. *J. Pers. Soc. Psychol.* **76**, 820–838 (1999).

12. Öhman, A., Flykt, A. & Esteves, F. Emotion drives attention: Detecting the snake in the grass. *Journal of Experimental Psychology: General* **130**, 466–478 (2001).

13. Kok, B. E. & Fredrickson, B. L. Upward spirals of the heart: Autonomic flexibility, as indexed by vagal tone, reciprocally and prospectively predicts positive emotions and social connectedness. *Biol. Psychol.* **85**, 432–436 (2010).

14. Mathews, A. & MacLeod, C. Cognitive Approaches to Emotion and Emotional Disorders. *Annu. Rev. Psychol.* **45**, 25–50 (1994).

15. Whitmer, A. J. & Gotlib, I. H. An attentional scope model of rumination. *Psychol. Bull.* **139**, 1036–1061 (2013).

16. Carl, J. R., Soskin, D. P., Kerns, C. & Barlow, D. H. Positive emotion regulation in emotional disorders: A theoretical review. *Clin. Psychol. Rev.* **33**, 343–360 (2013).

# Figures


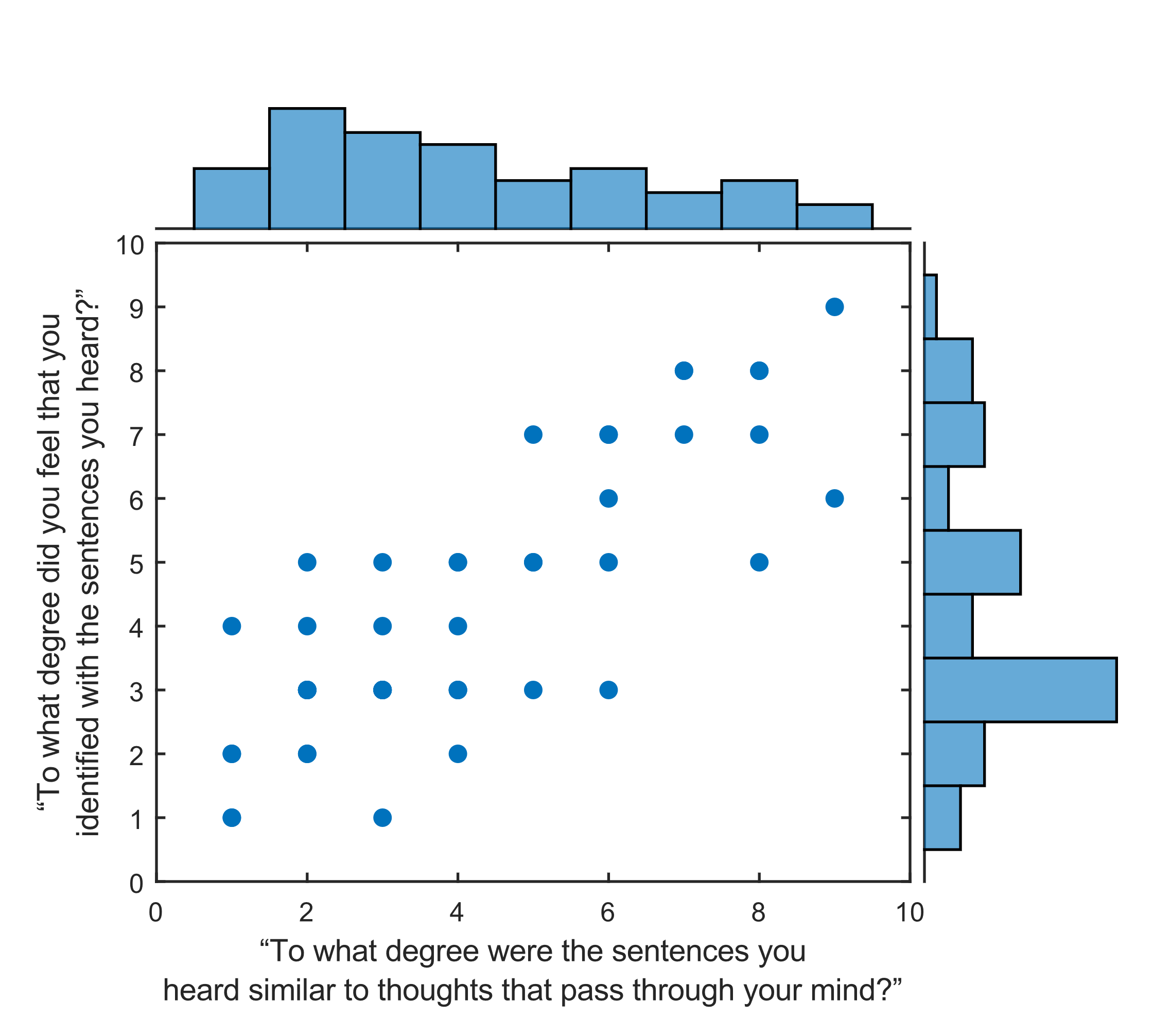


SM Figure 1. Scatter plot and histograms of identification and similarity ratings of STP stimuli (*r*(57) = .816, *p* < .001). Scale: 0 = “Not at all”, 10 = “Very much so”.

|  | **Table 1.** Descriptive statistics of questionnaires in Studies 1 & 2. SD = Standard Deviation, PTQ = Perseverative Thinking Questionnaire (repetitive negative thinking), PSWQ = Penn State Worry Questionnaire, CFQ = Cognitive Fusion Questionnaire, Public Health Questionnaire – 9 (depression symptom levels), BAI = Beck Anxiety Inventory. | | | | | | |
| --- | --- | --- | --- | --- | --- | --- | --- |
|  | **Questionnaire** | **Mean (SD)** | **Minimum** | **.25 Quantile** | **.50 Quantile** | **.75 Quantile** | **Maximum** |
| **Study 1** | PTQ | 22.44 (13.72) | 0 | 11 | 23 | 31 | 58 |
| (N = 48) | Brooding (RRS) | 9.88 (3.98) | 5 | 6 | 9 | 12 | 19 |
|  | PSWQ | 51.42 (12.86) | 30 | 43 | 53 | 62 | 76 |
|  | CFQ | 20.54 (9.77) | 7 | 12 | 20 | 28 | 49 |
|  | PHQ-9 | 5.18 (4.15) | 0 | 2 | 4.5 | 7 | 21 |
|  | BAI | 8.76 (9.51) | 0 | 2 | 6 | 12 | 46 |
|  |  |  |  |  |  |  |  |
|  | **Questionnaire** | **Mean (SD)** | **Minimum** | **.25 Quantile** | **.50 Quantile** | **.75 Quantile** | **Maximum** |
| **Study 2** | PTQ | 20.86 (13.63) | 0 | 10 | 19 | 29 | 57 |
| (N = 74) | Brooding (RRS) | 9.04 (3.08) | 5 | 7 | 8 | 11 | 18 |
|  | PSWQ | 51.40 (14.22) | 20 | 41 | 52 | 63 | 80 |
|  | CFQ | 20.40 (10.41) | 7 | 11 | 18.5 | 28 | 49 |
|  | PHQ-9 | 5.08 (3.66) | 0 | 2 | 4.5 | 7 | 18 |
|  | BAI | 8.90 (8.60) | 0 | 2 | 7 | 13 | 50 |

| **Table 2.** Indirect path coefficients, bootstrapped standard deviation, upper and lower confidence interval. PER = Positive Emotional Reactivity, RNT = Repetitive negative thinking, CFQ = Cognitive Fusion Questionnaire, D1B-Diff = Dichotic 1-Back Mean Difference Score. | | | | | | | | |
| --- | --- | --- | --- | --- | --- | --- | --- | --- |
|  |  |  |  | **Indirect path (a_1_·d_21_·b_2_)** | | | | |
| **X** | **M1** | **M2** | **Y** | **Beta** | **Boot SD** | **Upper CI** | **Lower CI** |  |
| PER | D1B-Diff | RNT | Depression | 0.08 | 0.12 | 0.39 | -0.10 |  |
| PER | D1B-Diff | RNT | Anxiety | 0.19 | 0.29 | 0.95 | -0.28 |  |
| PER | D1B-Diff | Brooding | Depression | 0.08 | 0.11 | 0.40 | -0.09 |  |
| PER | D1B-Diff | Brooding | Anxiety | 0.14 | 0.21 | 0.73 | -0.16 |  |
| PER | D1B-Diff | CFQ | Depression | 0.07 | 0.11 | 0.38 | -0.08 |  |
| PER | D1B-Diff | CFQ | Anxiety | 0.18 | 0.29 | 1.09 | -0.22 |  |
| PER | D1B-Diff | Worry | Depression | 0.03 | 0.06 | 0.25 | -0.03 |  |
| PER | D1B-Diff | Worry | Anxiety | 0.07 | 0.14 | 0.55 | -0.09 |  |

1. To identify outliers based on their deviation from the mean, RTs were log transformed for normality before calculating standard-deviations. Cut-offs were selected a priori based on (Price et al., 2015; Zvielli, Bernstein, & Koster, 2015). [↑](#footnote-ref-1)
